# NacNac-zinc-pyridonate mediated ε-caprolactone ROP†

**DOI:** 10.1039/d3dt03344a

**Published:** 2023-11-20

**Authors:** Jack W. J. Hughes, Dawid J. Babula, Findlay Stowers-Veitch, Kang Yuan, Marina Uzelac, Gary S. Nichol, Michael J. Ingleson, Jennifer A. Garden

**Affiliations:** a EaStCHEM School of Chemistry, University of Edinburgh Edinburgh EH9 3FJ UK j.garden@ed.ac.uk

## Abstract

Herein we report the synthesis, isolation and polymerisation activity of two new zinc compounds based on a 2,6-diisopropylphenyl (Dipp) β-diiminate (NacNac) ligand framework with zinc also ligated by an amidate (2-pyridonate or 6-methyl-2-pyridonate) unit. The compounds crystallised as either monomeric (6-Me-2-pyridonate derivative) or dimeric (2-pyridonate) species, although both were found to be monomeric in solution *via*^1^H DOSY NMR spectroscopy, which was supported by DFT calculations. These observations suggest that both complexes initiate ring-opening polymerisation (ROP) through a single-site monometallic mechanism. High molecular weight poly ε-caprolactone (PCL) was achieved *via* exogenous initiator-free ROP conditions with both catalysts. An increase in the 2-pyridonate initiator steric bulk (6-Me- *vs.* 6-H-) resulted in an improved catalytic activity, facilitating complete monomer conversion within 1 h at 60 °C. Pyridonate end-groups were observed by MALDI-ToF mass spectrometry, contrasting with previous observations for ^Dipp^NacNac-Zn acetate complexes (where no acetate end groups are observed), instead this more closely resembles the reactivity of ^Dipp^NacNac-Zn alkoxide complexes in ROP (where RO end groups are observed). Additional major signals in the MALDI-ToF spectra were consistent with cyclic PCL species, which are attributed to back-biting ring-closing termination steps occuring in a process facilitated by the pyridonate unit being an effective leaving group. To the best of our knowledge, these complexes represent the first examples of pyridonate, and indeed amidate, initated ROP.

## Abstract 2

Intermediate reactivity between carboxylate and alkoxide initiated ROP is achieved here by a newly reported class of amidate initiators; pyridonates, resulting in a mixture of cyclic and linear polyesters being produced.

## Graphical abstract

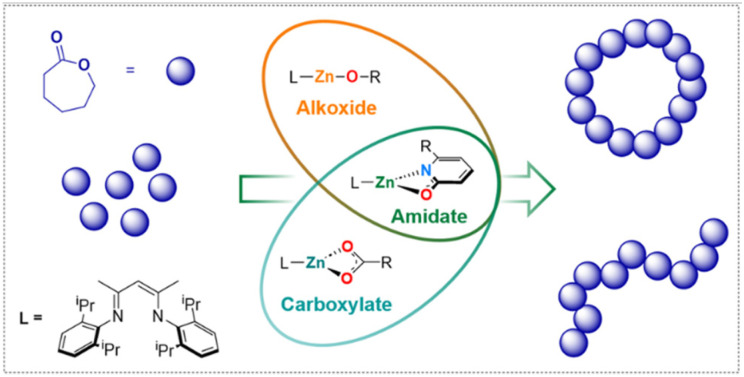

## Introduction

Petrochemical-derived plastics are essential to everyday life but have led to the accumulation of vast amounts of poorly recycled, non-biodegradable materials, which have made their way into all eco-systems.^1^ As a result, there has been a push for bioderived and biodegradable alternatives to traditional polymers such as poly(ethylene) (PE) and poly(propylene) (PP).^2,3^ These have come in large part in the form of polyesters such as poly(lactic acid) (PLA) and poly(caprolactone) (PCL), prepared from lactide and ε-caprolactone (ε-CL) respectively *via* ring-opening polymerisation (ROP). A variety of enzymatic^4^ and organocatalytic^5^ routes have been reported for the ROP of cyclic esters to polyesters, however organometallic ROP catalysts are increasingly attractive due to their high activity, good molecular weight control and well-understood mechanisms.^6,7^ Toxicity concerns^8^ with the current industry standard, stannous octoate, have limited the implementation of materials such as PLA and PCL in tailored applications in biomedicine.^9–11^ This has furthered efforts into exploring ROP catalysis based on more bio-compatible, Earth abundant metals such as sodium, aluminium, iron and zinc.^6^ These catalysts have shown promising activity and selectivity, in some cases out-performing the industry standard.^12–14^

**Fig. 1:**
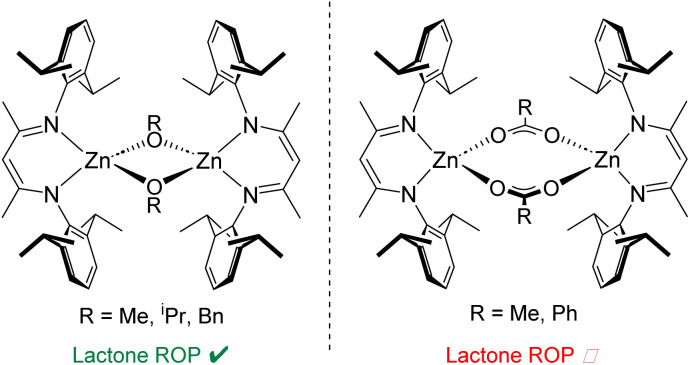
Select reported catalysts based on a ^Dipp^NacNac-Zn framework.^16,19^

Catalysts based on β-diiminate (NacNac) ligand frameworks have been studied extensively in polymerisation catalysis, including NacNac-Zn complexes.^15^ In the early examples by Coates and co-workers, a range of NacNac-Zn catalysts were prepared featuring monodentate alkoxide and bidentate carboxylate groups as co-ligands (Fig. 1).^16–20^ These studies included exploring the steric effect of the NacNac ligands upon polymerisation activity, highlighting the excellent efficiency of catalysts based on the bulky ^Dipp^NacNac (CH{C(Me)N-Dipp}_2_, Dipp = 2,6-diisopropylphenyl) framework. The efficacy of various initiating groups also was explored, both for lactone ROP and for the ring-opening co-polymerisation (ROCOP) of epoxides with CO_2_. NacNac-Zn catalysts with monodentate alkoxide co-ligands (which act as initiating groups) can facilitate both lactone ROP and epoxide/CO_2_ ROCOP, affording polymers with low dispersity (*Đ*) and good control of both number average molecular weight (*M*_n_) and chain-end fidelity.^16,18,20–22^ In contrast, NacNac-Zn catalysts with carboxylate (and structurally related sulfinate) co-ligands generally are only effective for ROCOP in which solution-state dimerisation effects are key to achieve good activity.^19,24,25^ Work by the groups of Coates, Schulz and Williams has shown acetate groups to be inefficient initiators for lactone ROP when combined with NacNac-Zn catalysts.^16,26,27^

Indeed, the work by Coates and co-workers suggests that the Zn-carboxylate moieties require transformation by trace impurities to form species in low amounts which in turn initiate ROP.^16^ While alkoxide and carboxylate co-ligands are ubiquitous in this field, co-ligands that combine features of both these co-ligand classes are much less explored, despite the potential to access unique reactivity. 2-Pyridonates are bidentate co-ligands with a conjugate acid of intermediate Brønsted acidity (*ca.* 11.70–12.45)^28^ between that of the conjugate acid of an alkoxide (*e.g.* O^i^Pr, p*K*_a_ of HO^i^Pr = 17.26, typically highly active ROP initiators) and a carboxylate (*e.g.* HOAc, p*K*_a_ = 4.76, characteristically poor initiators in ROP) in H_2_O.^29^ Therefore we were interested in installing these as co-ligands onto NacNac-Zn and determining how they functioned in the ROP of a cyclic ester. Herein we describe the synthesis of two new ^Dipp^NacNac-Zn complexes both featuring a bidentate pyridonate and show that this co-ligand is an effective initiating group towards ROP catalysis. While this positions 2-pyridonates closer to alkoxide co-ligands than carboxylate co-ligands in NacNac-Zn mediated ROP, the formation of a significant amount of cyclic polyesters confirm they represent a unique co-ligand class.

## Results and discussion

### Catalyst synthesis and characterisation

**Scheme 1:**
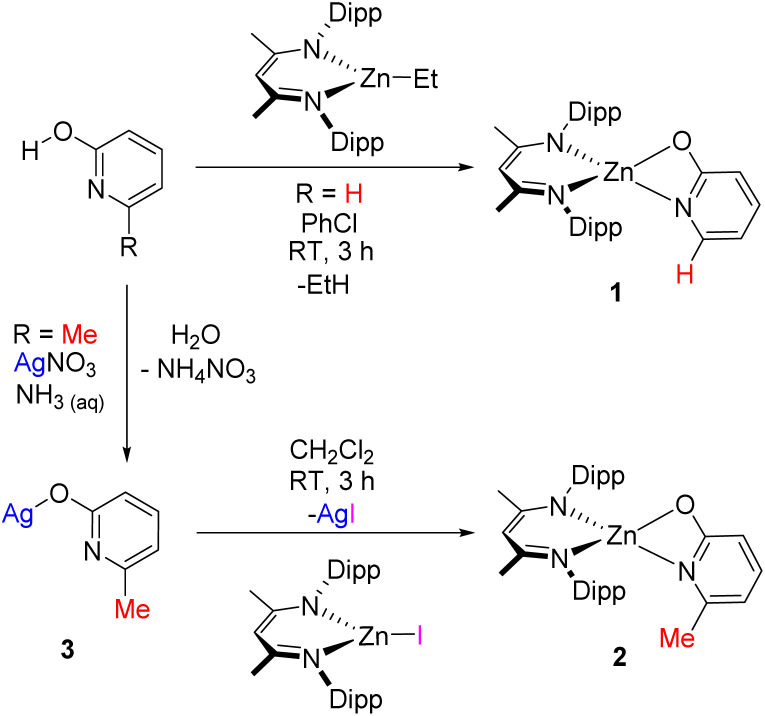
Synthetic routes to complexes 1 and 2 (the latter *via*3, which is presumably oligo-/poly-meric).

**Fig. 2:**
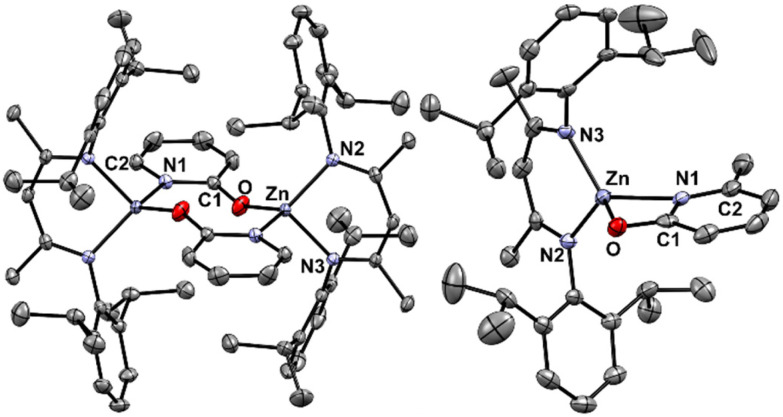
Molecular structures of complexes 1 (left) and 2 (right). H atoms and solvent omitted for clarity; ellipsoids are shown at the 30% probability level.

Complex 1 was synthesised *via* reaction of 2-pyridone with ^Dipp^NacNac-ZnEt, with an isolated yield of 40% following fractional recrystallisation. The methyl-substituted analogue 2 also could be formed by this route, albeit in a crude mixture, and in this case 2 could not be isolated cleanly by fractional crystallisation. Compound 2 instead was prepared *via* a salt-metathesis route, employing ^Dipp^NacNac-ZnI^30^ and the silver pyridonate 3. Insoluble AgI was formed as a by-product, which aided isolation of 2 and this approach afforded 2 in an 80% isolated yield (Scheme 1). Compound 3 is also novel and is presumably an oligo-/poly-meric material given its low solubility in common solvents – characterisation is given in the ESI.† Single crystals of 1 and 2 suitable for X-ray diffraction studies were grown from CH_2_Cl_2_. Complex 1 crystallised as a dimer; containing an 8-membered ring between two distorted tetrahedral zinc centres (Fig. 2). Complex 2, by comparison was monomeric, with a strained 4 membered metallacycle involving the distorted tetrahedral zinc centre. The crystallisation of 2 as a monomer was notable as no carboxylate NacNac-Zn(O_2_C-R) complexes have been reported as monomers with 4-membered rings; instead these dimerise to form an 8-membered ring (related to that seen for 1). This was the first indication of a significant disparity between carboxylates and these pyridonates in these NacNacZn systems. It should be noted that 2-pyridonates have been shown to form four membered metallacycles in other systems.^31^ The increased valency of nitrogen (present in the pyridonate) *versus* oxygen (in carboxylates) leads to the incorporation of more steric bulk proximal to the Zn–ligand bond, which will influence aggregation in both the solid and solution state. Indeed, work by Cundari, Holland and co-workers on NacNac-Fe complexes bearing *N-R*-amidinates (R = ^i^Pr) as co-ligands were observed to be solely monomeric, attributable to the *N*-steric bulk.^32^ It is likely that the greater steric crowding present using pyridonates (relative to carboxylates) helps disfavour dimerisation, in contrast to the analogous carboxylates where steric bulk can only be modified at the distal (relative to zinc) carbon position. An additional factor favouring dimerization with carboxylates will be the slightly larger O–C–O angle observed in [NacNac-Zn(O_2_C-R)] complexes (121–128°) relative to the N–C–O angles in 1 and 2 (*vide infra*).

The C–O bond lengths in both 1 and 2 (1.280(3) Å and 1.301(4) Å, respectively, Table 1) are intermediate between a single and a double C–O bond, and are greater than in the protonated ligands (1.250(0) Å and 1.262(5) Å respectively).^33,34^ Further, there was a reduction in C–N bond lengths for the pyridone unit (1.379(1) to 1.356(3) Å in 1, and 1.391(6) to 1.357(4) Å in 2), again relative to the protonated ligand, suggesting some multiple bond character. The lengthening of the CO bond and contraction of the CN bond in both instances indicates a significant degree of charge delocalisation in the amidate unit when bound to zinc, but with the oxypyridine form more dominate in 1 and 2 than in the protonated ligand (where the pyridone resonance form is more dominant). A narrow N1–Zn–O bond angle of 65.7(1)° was observed in 2, suggesting a significant degree of ring strain that is alleviated in 1 through dimerisation, which increases the N1–Zn–O bond angle to 113.3(3)°. In both complexes, a reduction in the O–C–N1 bond angle of the pyridone unit was observed relative to their protonated form, which was more pronounced in 2 (−5.5° *cf.* the protonated ligand). The reduction in the O–C–N bond angle upon metallation is in-line with other 4-membered pyridone metallacycles in the literature.^31,35^

Select relevant bond lengths and angles in complexes 1 and 2

**Table:** 

Bond	Bond angles/Å	
	1	2
Zn1–O1	1.896(2)	2.038(2)
Zn1–N1	2.026(9)	2.074(3)
Zn1–N2	2.0005(12)	1.942(3)
Zn1–N3	1.9923(12)	1.943(3)
O1–C1	1.280(3)	1.301(4)
N1–C1	1.356(3)	1.357(4)
N1–C2	1.355(4)	1.356(4)

**Table:** 

Bonds	Bond angles/°	
N1–Zn1–O1	113.3(3)	65.7(1)
Zn1–O1–C1	153.3(4)	91.6(2)
Zn1–N1–C1	122.0(5)	88.5(2)
N2–Zn1–N3	96.52(5)	99.41(12)
N1–C1–O1	118.5(4)	114.2(3)

The differing solid-state structures (monomeric *vs.* dimeric) meant that direct comparison of the effect of changing the co-ligand on the solid state structure of complexes 1 and 2 was not possible. Therefore the monomer and dimer structures were calculated in both cases at the B3PW91 level of theory using the LANL2DZ basis set for Zn and 6-311G(d,p) basis set for all other atoms in CH_2_Cl_2_ using a polarisation continuum model (PCM) at 298 K. A slight increase in the Zn–N bond length and decrease in Zn–O bond length was observed for the monomeric structure of 2 compared to 1 (Table S4†), however the majority of the bond lengths and angles were extremely similar. Comparison of the free energies of mono-/dimeric 1 and 2 suggest the dissolution of the dimer into two monomers is energetically favoured in both cases (by 24.2 and 36.9 kcal mol^−1^ respectively, Fig. S21†). The significantly energetically favoured conversion of the dimers into monomers was corroborated by ^1^H DOSY NMR spectroscopy. At the concentration of the polymerisation reactions (8.7 mM in catalyst, *vide infra*) in toluene-*d*_8_, 1 and 2 were observed to be monomeric. This was evidenced by a single diffusion coefficient, which was compared to a calibration plot in toluene-*d*_8_ (see Table S3 and Fig. S19 in ESI† for experimental set up and calibration), and gave estimated molecular weights of 572 and 581 g mol^−1^ for 1 and 2. These calculated values are within 2% error of the expected values for the monomeric complexes. Taken together, the DFT and DOSY NMR studies of 1 and 2 suggest that ROP takes place *via* a monometallic single-site mechanism. Analysis of the symmetry by ^1^H NMR spectroscopy (using the ^i^Pr methyl groups in ^Dipp^NacNac) for both 1 and 2 revealed *C*_2v_ solution symmetry as there were only two discrete environments observed (Fig. S1 and S6†). This suggests that the 4-membered metallacycles in 1 and 2 undergo ring-opening on the NMR time scale which is consistent with significant strain in the four membered metallacycle.

### Polymerisation studies

Complexes 1 and 2 were tested in the ROP of ε-caprolactone (ε-CL). Catalytic activity was investigated using a [catalyst] : [ε-CL] ratio of 1 : 100 between 20–60 °C in toluene; these are typical conditions for lactone ROP (Table 2).^36^ Significant polymerisation activity was observed for complex 2 at 60 °C, converting all ε-CL within 1 h (Table 2, entry 4). This was significantly faster than catalyst 1, which converted 64% of ε-CL under identical conditions and did not reach full conversion due to viscosity limitations (Table 2, entries 2 and 3). This trend was more pronounced at room temperature; complex 2 converted over seven times as much ε-CL in 4 h as 1 converted in 24 h (Table 2, entries 1 and 8). To confirm that ROP activity was due to complexes 1 and 2, zinc free control polymerisations with the two pyridone (2-pyridone for 1 and 6-methyl-2-pyridone for 2) ligands were conducted (Table 2, entries 4 and 9). These revealed 0% conversion after 4 h, suggesting that ROP activity was indeed due to zinc complexes 1 and 2 and not low quantities of free ligand.

**Table:** ROP of ε-CL catalysed by complexes 1 and 2

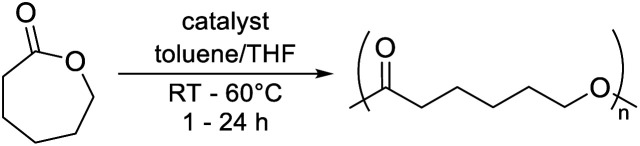								
Entry	Catalyst	Temperature	Time (h)	Conversion^a^ (%)	*M*_n_ (kDa)		*Đ* c	Initiation efficiency^d^ (%)
					Calculated^b^	Observed^c^		
1	1	RT	24	6	—	—	—	—
2	1	60 °C	1	64	7.4	58.8	1.10	12.4
3	1	60 °C	4	97	11.1	121.1	2.24	9.1
4^e^	**Pyridone**	60 °C	4	0	—	—	—	—

5	2	60 °C	1	>99	11.4	77.6	1.48	14.7
6	2	RT	1	2	0.2	9.5	—	2.5
7	2	RT	2	10	1.1	25.4	1.41	4.7
8	2	RT	4	43	4.9	45.8	1.47	11.2
9^e^	**Me-pyridone**	RT	4	0	—	—	—	—

10^f^	2	RT	1	0	—	—	—	—
11^f^	2	RT	4	17	2.0	22.8	1.23	9.1
12^f^	2	RT	23	81	9.3	51.0	1.32	19.1

a Conversion was calculated *ex situ* by ^1^H NMR spectroscopy.

b *M*_n calc_ of polymers calculated from monomer conversion; *M*_n calc_ = ([ε-CL]_0_/[Cat]_0_) × (% conversion of ε-CL) × 114.14, assuming 1 polymer chain per catalyst centre.

c *M*_n obs_ and *Đ* determined by size exclusion chromatography (SEC) using polystyrene standards in THF: values were corrected using a correction factor (0.56).^37^

d Calculated according to eqn (S1)–(S3).†

e Zinc free control reaction with the respective pyridone substrate.

f Performed in THF rather than toluene.

Greater than calculated *M*_n_ values were observed using both complexes as catalyst, which was attributed to poor initiation resulting in *k*_propagation_ > *k*_initiation_. The discrepancy between theoretical and experimental *M*_n_ was quantified by assigning an initiation efficiency value, which describes the theoretical % of active species which initiate ROP (eqn (S1)–(S3)†). While NacNac-Zn alkoxide complexes have been shown to exhibit high initiation efficiency, and therefore good *M*_n_ control, moving to less basic co-ligands (like carboxylate) results in a significant drop off in initiation efficiency, as evidenced in the work by Coates and co-workers (*vide supra*).^16^ The poor initiation efficiency of 1 resulted in very high MW PCL which formed a gel and hindered efficient mixing. This prevented full conversion from being achieved (Table 2, entry 3). An increase in *Đ* from 1.10 to 2.24 was noted (Table 2, entries 2 and 3), attributed to transesterification side reactions at high conversions.

Contrastingly, complete conversion was achieved with 2 accompanied by notably lower dispersity without mixing issues due to gelation (Table 2, entry 5). The slightly higher initiation efficiency for 2 is tentatively assigned to the greater steric encumbrance from the methyl group, favouring ring opening and forming a κ^1^-bound pyridonate. This is presumably required to enable the initiation step, which may proceed *via* monomer coordination. In any case, the initiation efficiency of both catalysts was noted to be poorer than the NacNac-Zn alkoxide complexes, which have demonstrated good control over *M*_n_ and *Đ*.^16^

**Fig. 3:**
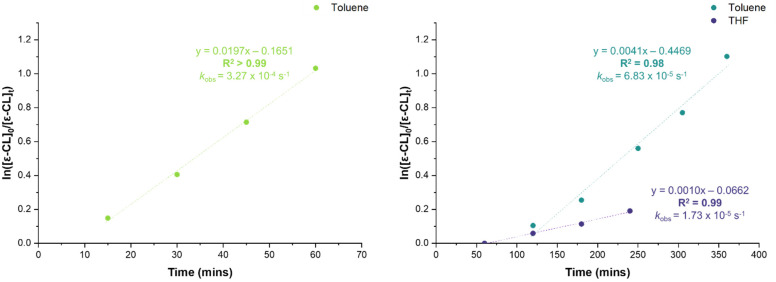
1st order kinetic plot of ε-CL consumption by complexes 1 (60 °C, left, green) and 2 (RT, right, blue and purple).

Kinetic studies revealed that both complexes undergo an induction period, which was significantly reduced at elevated temperatures (Fig. 3). Induction periods have been observed for organometallic zinc-catalysed ROP and have been shown to vary with temperature, solvent and monomer.^36,39–41^ Post-induction, polymerisation proceeds *via* first order kinetics with respect to [ε-CL]. The near 4-fold reduction in *k*_obs_ in THF *cf.* toluene is proposed to be due to coordinative competition between the THF solvent and the monomer, a phenomenon that has previously been observed in the literature.^36,38,42,43^ Note, with this bulky ^Dipp^NacNac ligand the formation of 5-coordinate zinc complexes has extremely limited precedence therefore we disfavour their intermediacy in this process. Instead, we support the opening of the four membered strained metallacycle in monomeric 1 and 2 as a key step forming a three coordinate NacNac species that then binds an equivalent of monomer. We propose that this three coordinate zinc species is bonded through the oxygen of the pyridonate ligand, and that the increased sterics of 2 results in an increased propensity to ring-open the metallacycle due to steric repulsion, which could explain the increased rate relative to 1.

### End-group elucidation

**Fig. 4:**
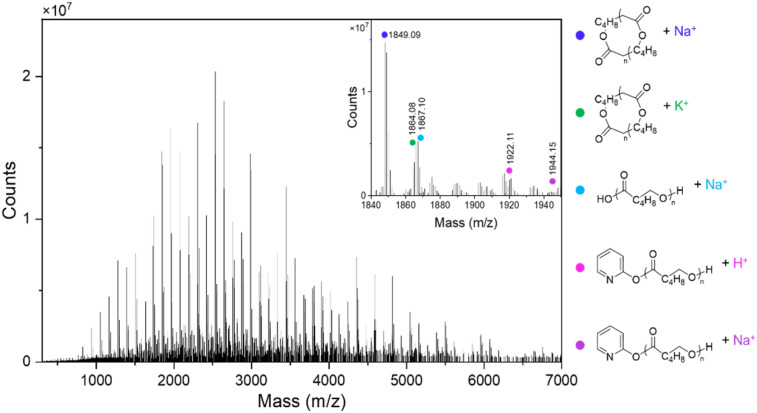
MALDI-ToF MS of PCL made in the presence of complex 1 after 1 h at 60 °C (Table 2, entry 2), with a highlighted region shown to illustrate the polymeric species present.

The high MW of the PCL produced by both catalysts made elucidation of end-groups by ^1^H NMR spectroscopy difficult, especially given the high MW at low polymer conversions (Table 2, entries 6 and 7). Therefore, matrix assisted laser desorption ionisation time-of-flight mass spectrometry (MALDI-ToF MS) analysis was used on both purified and crude PCL samples formed using catalysts 1 and 2. Major peaks corresponding to cyclic PCL were detected, with some minor peaks attributed to both pyridonate-capped chains and hydroxyl-capped chains (Fig. 4). This is strikingly different to that of NacNac-zinc acetate mediated lactone ROP reported in the literature, where polymer chains featuring acetate end groups have not been reported; this is presumably due to the greater nucleophilicity of the Zn-pyridonate unit enabling it to act as an initiator.

While the cyclic PCL signals may be overestimated when compared to their linear counterparts,^44^ their formation warrants discussion. This may occur through intramolecular back-biting/ring-closure termination reactions during polymerisation. It is possible that the leaving ability of the pyridonate group (consistent with the p*K*_a_ of the pyridone units) facilitates this step, by enabling displacement of the relatively stable (compared to alkoxide) pyridonate anion and cyclisation of the PCL chain (refer to Fig. S14† for a proposed simplified schematic). Indeed, phenoxo-imine ligands complexed with group 1 metals have illustrated this ring-closing phenomenon, which also was attributed to the formation of more stable (than alkoxide) anionic leaving groups.^45,46^ While few studies have directly determined the p*K*_a_ of the Schiff bases used in complexation, studies on structurally similar Schiff bases have revealed p*K*_a_ values around 11, similar in magnitude to that of the pyridonates utilised herein.^47^ Cyclic polyesters have been shown to form during the ROP of cyclic esters with main group metal complexes of the *TrenSal* ligand, as reported previously by some of us.^45^ Comparison of the *calculated* p*K*_a_ values for the TrenSal ligand and the pyridone ligands reported herein reveal they are between 9.11–11.91 in H_2_O, which tentatively supports a p*K*_a_ based argument for cyclisation.^48^ NacNac-Zn alkoxide initiators, where the conjugate acid of the co-ligand typically has a much higher p*K*_a_, have not been reported to undergo analogous back-biting reactions to form cyclic polymers, and instead retain the alkoxide end-groups on linear polymer chains.^23,49^ In any case, the variety of PCL end-groups observed by MALDI-ToF is indicative of poor/stochastic initiation.

MALDI-ToF MS analysis of PCL samples prepared with 2, purified by quenching in wet CDCl_3_ and then precipitating in excess cold acidified MeOH revealed signals attributed to methoxy end groups (Fig. S13†) that were not observed in the crude samples. We attribute this to a combination of transesterification of the polymer end-groups and the ring-opening of macrocyclic caprolactone polymers. This phenomenon has been observed previously by Mehrkhodavandi and co-workers for PLA samples synthesised through zinc-catalysed ROP, that were quenched in wet chloroform and precipitated in cold MeOH,^50^ and by Sarkar, Chandrasekhar, Panda and co-workers, who used methanol to *quench* lactone polymerisations.^51^ In both instances, MeO end-groups were observed by either MALDI-ToF or ^1^H NMR spectroscopy, suggesting that this is not a feature of our catalytic system but rather an implication of the quenching and/or precipitation method.

## Conclusions

Two new ^Dipp^NacNac-Zn complexes with a pyridone-based co-ligand were synthesised and characterised in both the solid and solution-state. These complexes were active in the room temperature ROP of ε-CL to afford high MW PCL in the absence of exogenous co-initiators. Despite their different aggregation states in the solid state, both complexes are dominated by the monomeric form in the solution state under the polymerisation conditions. Therefore, these complexes are expected to act as single-site monometallic catalysts. MALDI-ToF MS analysis of the polymer products revealed a variety of end-groups with the formation of significant cyclic PCL species detected. This is distinct to ^Dipp^NacNac-Zn-alkoxide derivatives and was attributed to a side-reaction involving back-biting of the growing polymer chains, facilitated by the relatively low (*e.g.* compared to ^i^PrOH) p*K*_a_ values of the pyridone groups enabling cleavage of the C(O)–O_pyridone_ bond and formation of a pyridonate leaving group. Further, another series observed in the MALDI-ToF spectrum suggests that the pyridonate moiety can initiate ROP, furnishing pyridone end-capped PCL chains. These observations combined suggest that ROP proceeds by the ring-opening of the 4-membered Zn–N–C–O metallacycle to form a ^Dipp^NacNac-Zn-aryloxide that is three coordinate at zinc and thus can bind a molecule of monomer to initiate polymerisation. This work shows that amidate groups can facilitate ROP of lactones under mild conditions, with distinct behaviour relative to acetate (which requires an initiator) and alkoxide (which forms no cyclic polymer), which suggests that amidate co-ligands warrant further exploration in other polymerisations.

## Experimental

### General experimental details

All manipulations requiring inert conditions were performed under an argon atmosphere using standard Schlenk techniques or in a glove box. ^Dipp^NacNac-ZnI^30^ and ^Dipp^NacNac-ZnEt^52^ were synthesised using previously reported procedures. All reagents and solvents were obtained from Sigma-Aldrich, Fischer Scientific, Honeywell or Acros Organics and were used without further purification unless described otherwise. Dry THF, toluene and hexane were collected from a solvent purification system (Innovative Technologies), and stored over activated 4 Å molecular sieves under an argon atmosphere. Benzene-*d*_6_, chloroform-*d*, dimethylsulfoxide-*d*_6_ and toluene-*d*_8_ solvents were degassed by three freeze–pump–thaw cycles and stored over activated 4 Å molecular sieves under an argon atmosphere. ε-Caprolactone (ε-CL) was purified by vacuum distillation and was stored over activated 4 Å molecular sieves in the freezer. All manipulations involving silver compounds were kept in the dark with aluminium foil wrapping. ^1^H, ^13^C and 2D NMR (DOSY) spectra were recorded on Bruker AVA400, PRO500, AVA500 and AVA600 spectrometers at 298 K at 400 MHz, 500 MHz and 600 MHz and referenced to the residual solvent peaks (^1^H: *δ* 7.16 (C_6_D_6_), *δ* 7.26 (CDCl_3_), *δ* 2.50 ((CD_3_)_2_SO) and *δ* 2.08 (CD_3_C_6_D_5_)). The multiplicity of the signals are indicated as “s”, “d”, “t” “q” “pent”, “sept” or “m” for singlet, doublet, triplet, quartet, pentet, septet or multiplet, respectively. The reported DOSY masses (to the nearest whole number) and aggregation states were determined by comparison to a calibration plot made with a range of standards (adamantane, 2-phenylpyridine, pyrene, tri(*o*-tolyl)-phosphine, tetraphenylnaphthalene, 2,2′-bis(diphenylphosphino)-1,1′-binaphthyl (BINAP)) with molecular weights varying from 136.2 to 622.7 g mol^−1^ in toluene-*d*_8_. More details are found in the ESI.† Elemental microanalyses of the silver salts for carbon, hydrogen, and nitrogen were performed by Elemental Microanalysis Ltd. SEC analyses of the filtered polymer samples were carried out in GPC grade THF at a flow rate of 1 mL min^−1^ at 35 °C on a 1260 Infinity II GPC/SEC single detection system with mixed bed C PLgel columns (300 × 7.5 mm). Suitable crystals of 1 or 2 were selected and mounted on a MITEGEN holder in oil on a Xcalibur, Eos diffractometer. The crystal was kept at 120.01(11) K during data collection. Using Olex2,^53^ the structure was solved with the SHELXT^54^ structure solution program using Intrinsic Phasing and refined with the SHELXL^55^ refinement package using *Least Squares* minimisation. Further experimental and refinement details are given in the CIF-files. CCDC 2285019–2285020 contains the supplementary crystallographic data for this paper.† MALDI-ToF MS analyses were performed using a Bruker Daltonics UltrafleXtreme™ MALDI-ToF/ToF MS instrument in either *linear* or *reflectron* mode. MALDI-ToF samples were made up either in a volume ratio of (a) 2 : 2 : 1 of polymer (10 mg mL^−1^), α-cyano-4-hydroxycinnamic acid (CHCA) (10 mg mL^−1^) and NaI (ionising agent, 10 mg mL^−1^) in THF or (b) 5 : 15 : 1 of polymer (10 mg mL^−1^), 2,5-dihydroxybenzoic acid (20 mg mL^−1^) and lithium iodide (10 mg mL^−1^) in THF. A droplet (2 μL) of the resultant mixture was spotted on to the sample plate and submitted for MALDI-ToF MS analysis.

### Synthesis of 3

In a 100 mL round bottom flask, AgNO_3_ (6.6 mmol, 1.12 g) was added to 20 mL of distilled water with stirring, followed by 2-hydroxy-6-methylpyridine (6 mmol, 0.57 g). NH_3 (aq)_ (11%, 6 mol L^−1^) in H_2_O was added dropwise with stirring until a neutral pH was achieved, affording an off-white suspension. The off-white suspension was filtered and washed, in order, with H_2_O (10 mL), EtOH (10 mL) and Et_2_O (10 mL). The resulting off-white solid was dried *in vacuo* (<0.28 mbar) at 50 °C for 5 h (1.22 g, 5.76 mmol, 48%).

^1^H NMR (500 MHz, (CD_3_)_2_SO, 323 K): *δ* 7.23 (t, 1H, Ar*H*), 6.19 (d, 1H, Ar*H*), 6.16 (d, 1H, Ar*H*), 2.37 (s, 3H, CH_3_).

^13^C{^1^H} NMR (126 MHz, (CD_3_)_2_SO, 323 K): *δ* 169.24, 153.76, 138.76, 112.39, 106.22, 25.96.

Analytical data (%), calculated: C, 33.37; H, 2.80; N, 6.49; found: C, 33.42; H, 2.79; N, 6.47.

### Synthesis of 1

2-Pyridone (0.24 g, 2.5 mmol) was dissolved in dry PhCl (5 mL) and heated gently to fully dissolve the solid. In a separate flask ^Dipp^NacNac-ZnEt (1.28 g, 2.5 mmol) was dissolved in dry PhCl (5 mL) and both solutions were stirred separately for 5 minutes. The ^Dipp^NacNac-ZnEt solution was then added to the 2-pyridone solution dropwise. Upon addition the reaction mixture turned cloudy and began to effervesce. This solution was left to stir for 3 h at RT, after which the solution was concentrated under vacuum and placed in the freezer at −30 °C. After 16 h, a white powder formed which was isolated *via* filtration (0.57 g, 0.99 mmol, 40%).

^1^H NMR (500 MHz, CD_2_Cl_2_, 298 K): *δ* 7.26 (m, 1H, Ar*H*), 7.23 (m, 1H, Ar*H*), 6.19 (m, 1H, Ar*H*), 6.15 (m, 1H, Ar*H*), 4.99 (s, 1H, NC(Me)C*H*), 3.20 (sept, 1H, C*H*Me_2_ (^i^Pr)), 1.75 (s, 6 H, ArC*H*_3_), 1.19 (d, 12H, CH*Me*_2_), 1.08 (d, 12H, CH*Me*_2_).

^13^C{^1^H} NMR (126 MHz, CD_2_Cl_2_, 298 K): *δ* 172.98, 169.22, 144.27, 143.43, 142.39, 140.80, (134.12, 129.81, 128.54 and 126.56, residual PhCl solvent) 125.49, 123.49, 110.92, 109.67, 94.35, 27.95, 24.08, 23.75, 23.43.

APPI-MS: *m*/*z* [M]^+^: 575.2847 calculated [M]^+^: 575.2878.

### Synthesis of 2

Ag-6-Methylpyridonate (3, 331 mg, 1.64 mmol) was added to a Schlenk flask followed by ^Dipp^NacNac-ZnI (1.00 g, 1.64 mmol). Anhydrous CH_2_Cl_2_ (20 mL) was subsequently added to afford a cloudy yellow solution. The reaction was stirred in the dark at room temperature for 3 h, after which time stirring was stopped and the suspended solids were left to settle. The solution was filtered and the filtrate retained. CH_2_Cl_2_ was removed *in vacuo* from the filtrate to afford an off-white powder (0.766 g, 1.31 mmol, 80%). All crystals grown (from CH_2_Cl_2_ or hexane) were filtered and dried *in vacuo* at 50 °C for 6 h prior to NMR analysis and subsequent catalytic testing.

^1^H NMR (500 MHz, CD_3_C_6_D_5_, 298 K): *δ* 6.73–6.70 (m, 1H, Ar*H*), 5.88 (d, 1H Ar*H*, *J* = 5 Hz), 5.78 (d, 1H Ar*H*, *J* = 5 Hz), 4.87 (s, 1H, NC(Me)C*H*), 3.38 (sept, 1H, C*H*Me_2_ (^i^Pr)), 2.02 (s, 3H, ArC*H*_3_), 1.68 (s, 6 H, NC(*Me*)CH), 1.21 (d, 12H, CH*Me*_2_), 1.17 (d, 12H, CH*Me*_2_).

^13^C{^1^H} NMR (126 MHz, CD_3_C_6_D_5_, 298 K): *δ* 174.29, 169.70, 153.02, 144.21, 142.93, 141.39, 126.56, 124.31, 109.33, 108.88, 95.39, 28.76, 25.14, 24.74, 24.09, 23.14.

APPI-MS: *m*/*z* [M]^+^: 590.31131. Calculated [M]^+^: 590.30833.

### General polymerisation set up

In the glovebox, an air-tight 7 mL Supelco® glass vial was charged with 1 eq. of either 1 or 2 under argon and dissolved in dry solvent (toluene or THF, stored for >24 h over activated 4 Å molecular sieves). Another air-tight 7 mL Supelco® glass vial was charged with 100 eq. of ε-CL and dry solvent. Both vials were stirred at the target temperature for 5 minutes, after which time the monomer solution was transferred to the catalyst solution to start the polymerisation (the resultant polymerisation mixture has a monomer concentration of 0.87 M). The reaction was stirred at 300 rpm using a magnetic stirrer bar. After the desired amount of time, aliquots were taken from the reaction mixture and quenched in wet CDCl_3_ prior to ^1^H NMR analysis. Monomer conversion was calculated according to known monomer (4.15 ppm) and polymer (4.25 ppm) CH_2_ peaks in CDCl_3_. For PCL precipitated in MeOH, the reaction samples quenched in chloroform were dried using compressed air/nitrogen to remove all solvent. The crude PCL was then dissolved in a small amount (1–5 drops) of CHCl_3_ and added dropwise with stirring to an excess of cold (<0 °C) MeOH weakly acidified with HCl (approx. 2 drops of 12 M HCl per 50 mL of MeOH). The white precipitate was filtered and dried under air to give purified PCL.

## Author contributions

J. A. G. and M. J. I. supervised this project, and J. W. J. H. wrote the manuscript and performed the bulk of the reactions, with editing and revisions by both J. A. G. and M. J. I. Crystals were obtained by D. J. B. and solved by M. U. and G. S. N. 1 was tested by D. J. B. and F. S. and all computational calculations were performed by K. Y.

## Conflicts of interest

There are no conflicts to declare.

## Supplementary Material

DT-052-D3DT03344A-s001

DT-052-D3DT03344A-s002
