# Supplementary material for: NacNac-zinc-pyridonate mediated ε-caprolactone ROP
Source: Dalton Trans. 2023 Nov 20;52(47):17767–75. doi: 10.1039/d3dt03344a (PMC10696559; doi:10.1039/d3dt03344a)
Supplement: DT-052-D3DT03344A-s001 [file DT-052-D3DT03344A-s001.pdf]

Electronic Supplementary Material (ESI) for Dalton Transactions.
This journal is © The Royal Society of Chemistry 2023

Supporting Information

NacNac-zinc-pyridonate mediated ε-caprolactone ROP

Jack W. J. Hughes, Dawid J. Babula, Findlay Stowers-Veitch, Kang Yuan, Marina Uzelac, Gary S. Nichol, Michael J. Ingleson* Jennifer A. Garden*[a]

[a]*EaStCHEM School of Chemistry, University of Edinburgh, Edinburgh, EH9 3FJ, UK*

**E-mail: j.garden@ed.ac.uk*

1. Characterisation of complex **1** 3

Figure S1. ^{1}H NMR spectrum of complex **1** in CD_2Cl_2 (298 K) 3

Figure S2. $^{13}C\{^1H\}$ NMR spectrum of complex **1** in CD_2Cl_2 (298 K, * = PhCl). N.B. CH_2Cl_2 portion of the spectra removed for clarity 3

Figure S3. DOSY NMR spectra of **1** in $CD_3C_6D_5$ (298 K) at polymerisation relevant concentration 4

2. Characterisation of silver salt **3** 5

Figure S4. ^{1}H NMR spectrum of **3** in $(CD_3)_2SO$ (323 K) 5

Figure S5. ^{13}C NMR spectrum of **3** in $(CD_3)_2SO$ (323 K). 6

3. Characterisation of complex **2** 7

Figure S6. ^{1}H NMR spectrum of **2** in $CD_3C_6D_5$ (298 K) 7

Figure S7. $^{13}C\{^1H\}$ NMR spectrum of **2** in $CD_3C_6D_5$ (298K) 7

Figure S8: DOSY NMR spectrum of **2** in $CD_3C_6D_5$ (298K) 8

4. ROP of *ε*-CL in toluene/THF at RT to 60 °C catalysed by complexes **1 - 2**. 9

Table S1. ε-CL ROP data 9

5. MALDI-ToF spectra 10

Figure S9. MALDI-ToF MS analysis of crude PCL made in the presence of **1** after 4 h in toluene at 60 °C (table 2, entry 3). 10

Figure S10. MALDI-ToF MS analysis of crude PCL made in the presence of **1** after 24 h in toluene at 60 °C. 10

Figure S11. MALDI-ToF MS analysis of crude PCL made in the presence of **2** after 2 h in toluene at RT (table 2, entry 7). MALDI-ToF preparation method b) was used. 11

Figure S12. MALDI-ToF MS analysis of crude PCL made in the presence of **2** after 2 h in THF at RT. MALDI-ToF preparation method b) was used. 11

Figure S13. MALDI-ToF MS analysis of purified PCL (cold MeOH/H^+) made in the presence of **2** after 4 h in toluene at RT. MALDI-ToF preparation method b) was used 12

6. Proposed, simplified scheme for ring closure 12

Figure S14. Proposed ring-closure step using a simplified schematic: cyclisation of the PCL chain liberating the pyridonate anion to reform the catalytic species 12

7. Example SEC traces 13

Figure S15. Example SEC trace of crude PCL generated in the presence of **1** in toluene at 60 °C (1 h) (table 2, entry 2) 13

Figure S16. Example SEC trace of crude PCL generated in the presence of **1** in toluene at 60 °C (4 h) (table 2, entry 3) 13

Figure S17. Example SEC trace of crude PCL generated in the presence of **2** in toluene at RT (2 h) (table 2, entry 7) 14

Figure S18. Example SEC trace of crude PCL generated in the presence of **2** in THF at RT (2 h). 14

8. Equations 14

9. Crystal refinement data 15

Table S2. Crystal data and structure refinement for complex **1** (co-crystallised out of CH_2Cl_2) and **2** 15

10. DOSY parameters and instrumentation 16

Table S3. Diffusion coefficients of standards in toluene-d_8 solution compared to their molecular weights. 17

Figure S19. Plot of Log(D) vs. log(MW) constructed from the ^{1}H DOSY NMR data of adamantane, 2-phenylpyridine, pyrene, tri(o-tolyl)-phosphine, tetraphenylphthaline and 2,2′-bis(diphenylphosphino)-1,1′-binaphthyl (BINAP) standards in toluene-d_8. 17

11. Computational details for the structures of **1** and **2** 18

Figure S20. Monomeric computed structures of **1** (left) and **2** (right) 18

Table S4. Bond lengths and angles calculated for monomeric **1** and **2** 18

Figure S21: Calculated gain in free energy upon dissolution of **1** and **2** into their monomeric species 19

12. References 32

1. Characterisation of complex **1**

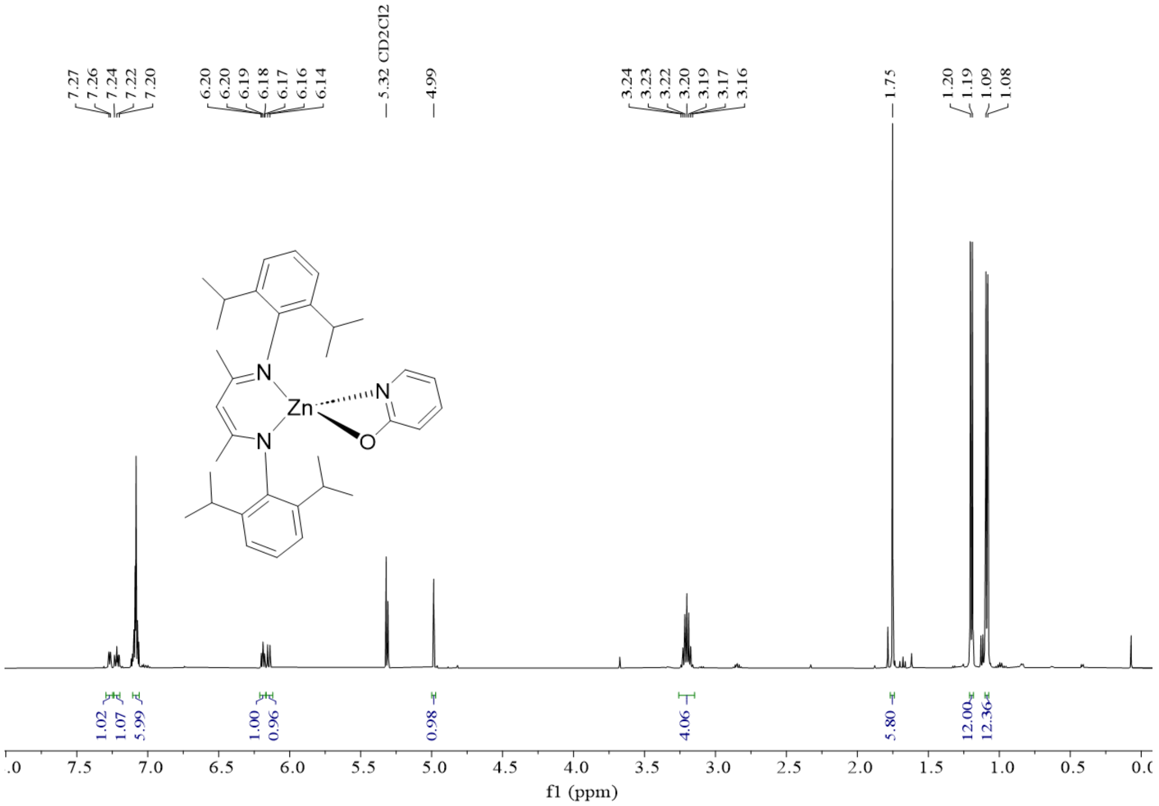

Figure S1. *^{1}H NMR spectrum of complex **1** in CD_2Cl_2 (298 K)*

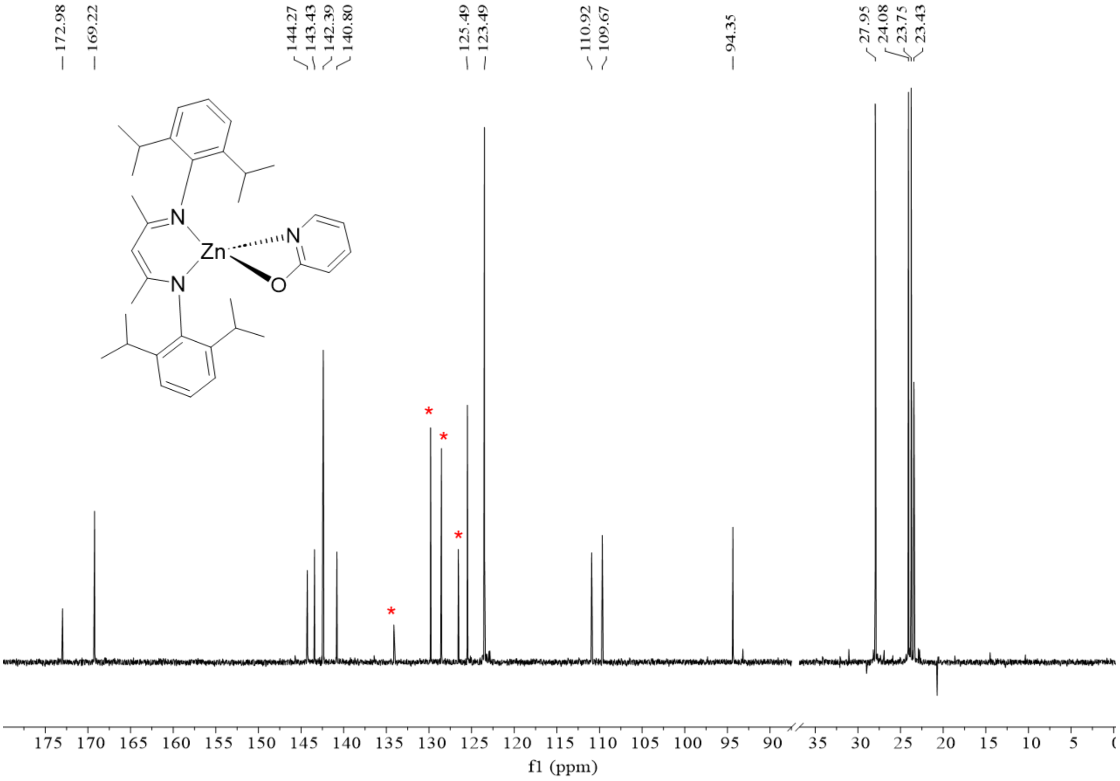

Figure S2. *$^{13}C\{^{1}H\}$ NMR spectrum of complex **1** in CD_2Cl_2 (298 K, * = PhCl). N.B. CH_2Cl_2 portion of the spectra removed for clarity*

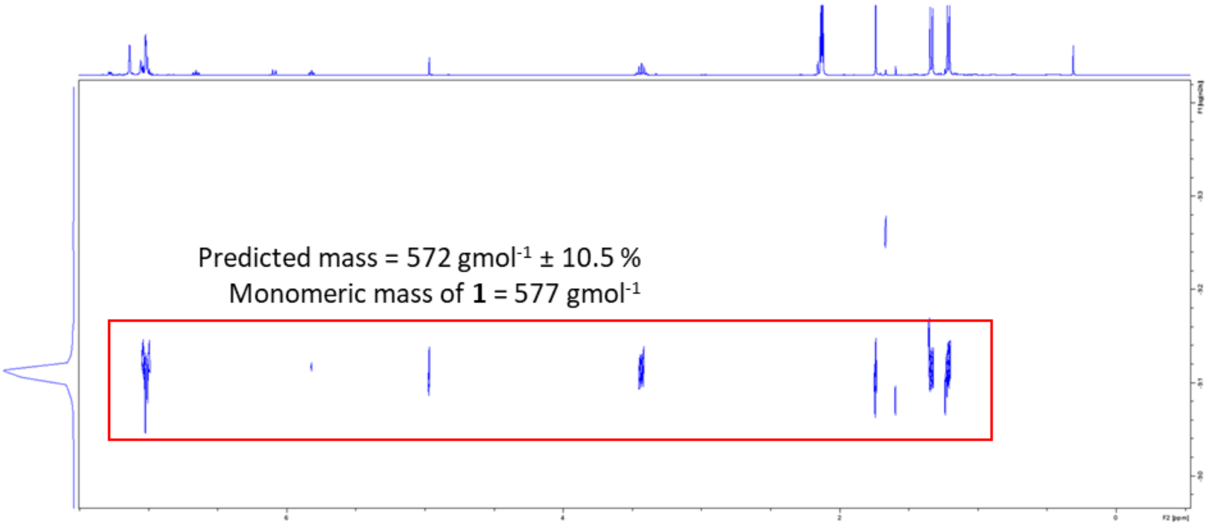

Figure S3. *DOSY NMR spectra of **1** in $CD_3C_6D_5$ (298 K) at polymerisation relevant concentration*

2. Characterisation of silver salt **3**

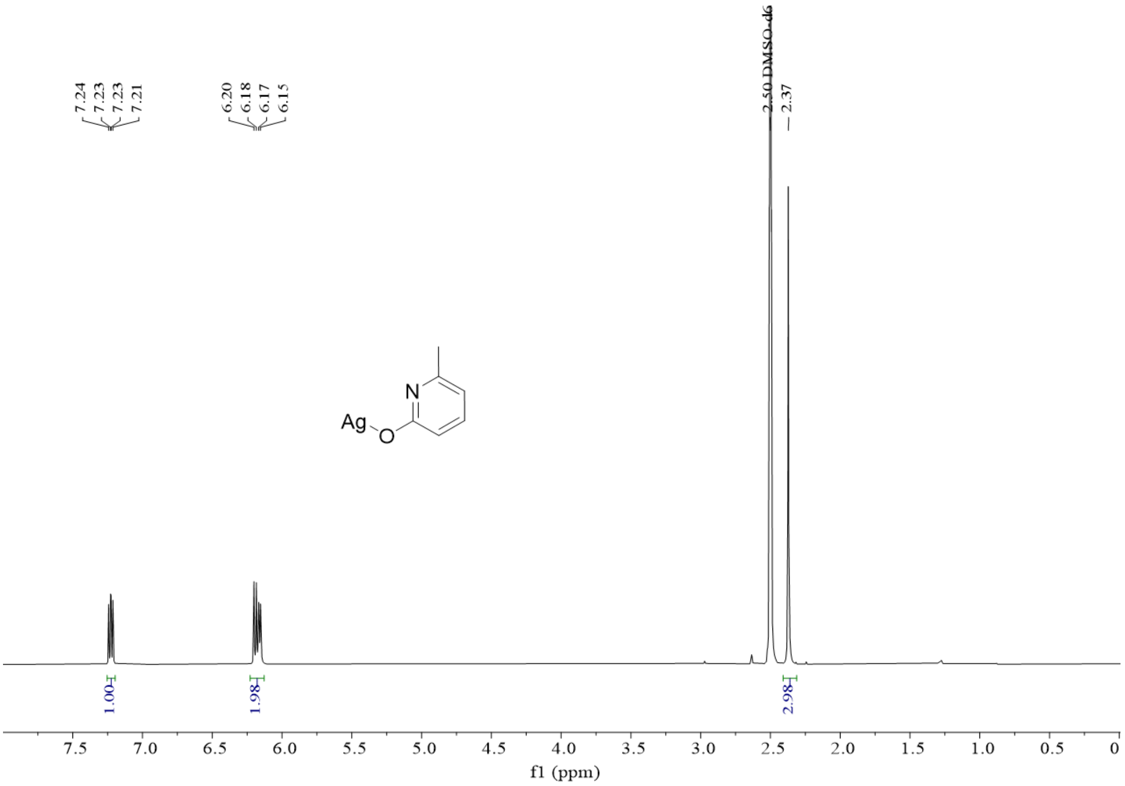

Figure S4. *^{1}H NMR spectrum of **3** in $(CD_3)_2SO$ (323 K)*

A marked downfield shift in proton resonance was noted *c.f.* the free pyridone in DMSO-d_6.[3] Note this compound is poorly soluble, hence NMR spectra were recorded at higher temperatures where the solubility is somewhat improved.

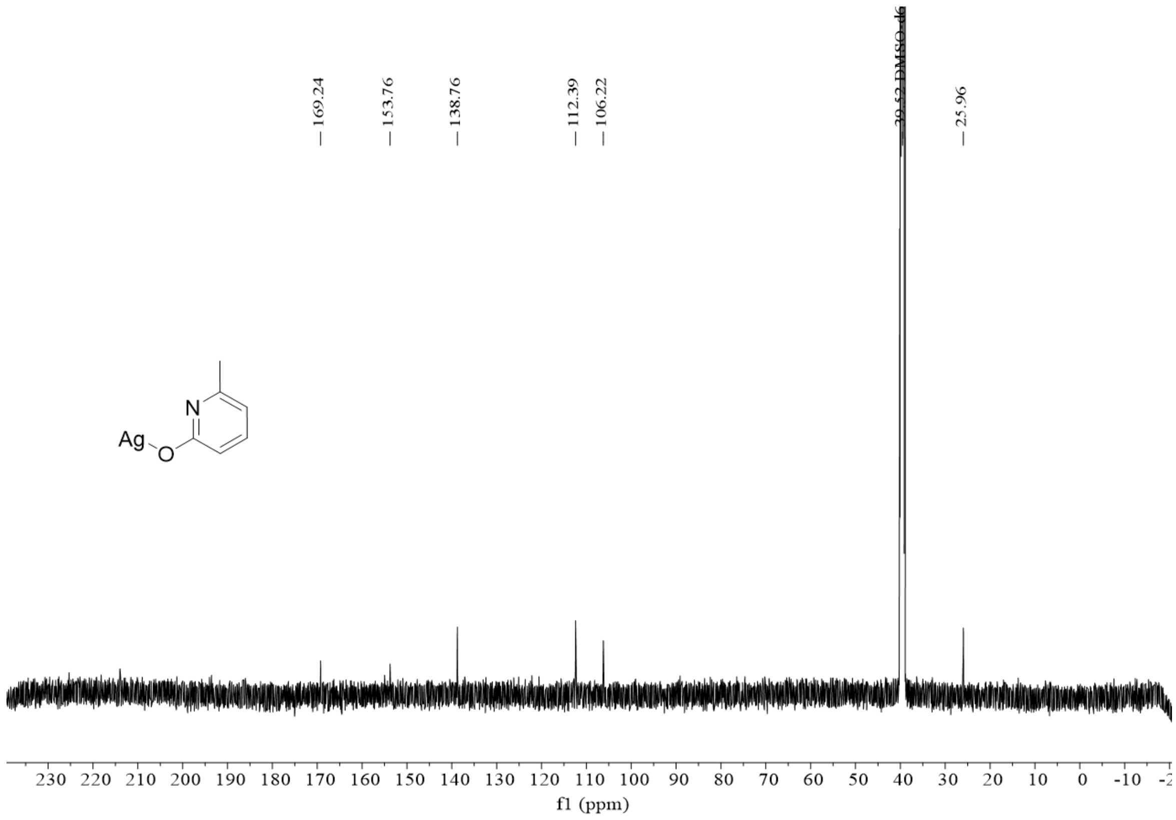

Figure S5. *^{13}C NMR spectrum of **3** in $(CD_3)_2SO$ (323 K).*

3. Characterisation of complex **2**

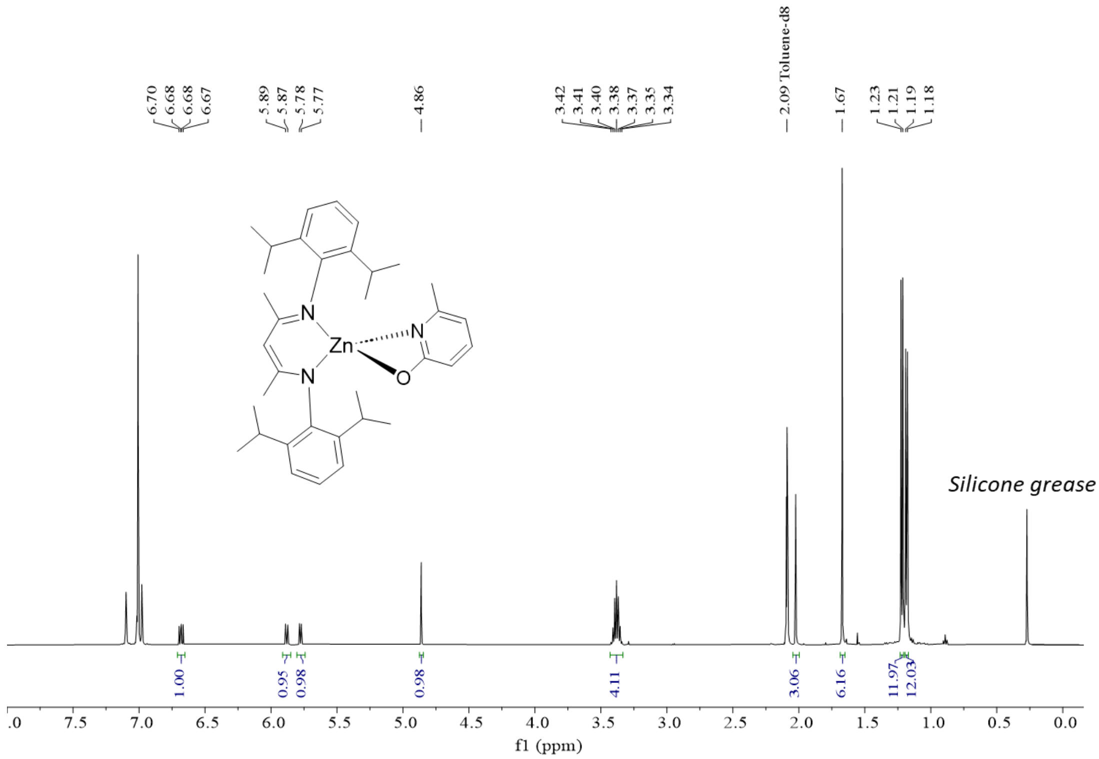

Figure S6. *^{1}H NMR spectrum of **2** in $CD_3C_6D_5$ (298 K)*

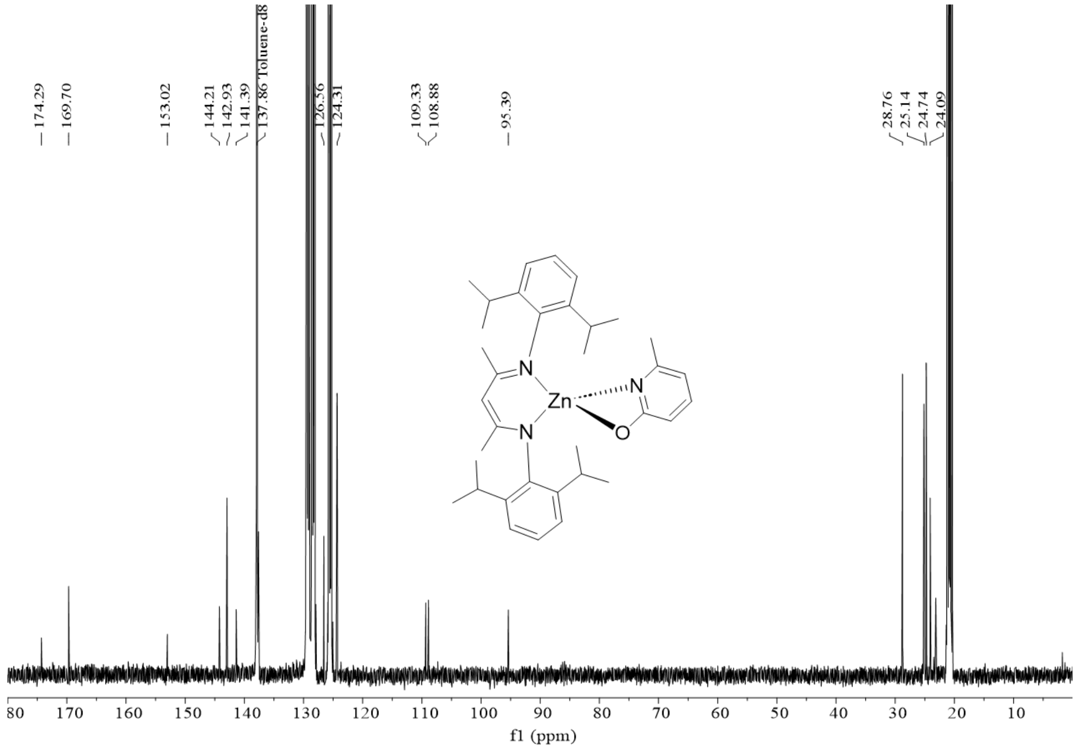

Figure S7. *$^{13}C\{^1H\}$ NMR spectrum of **2** in $CD_3C_6D_5$ (298K)*

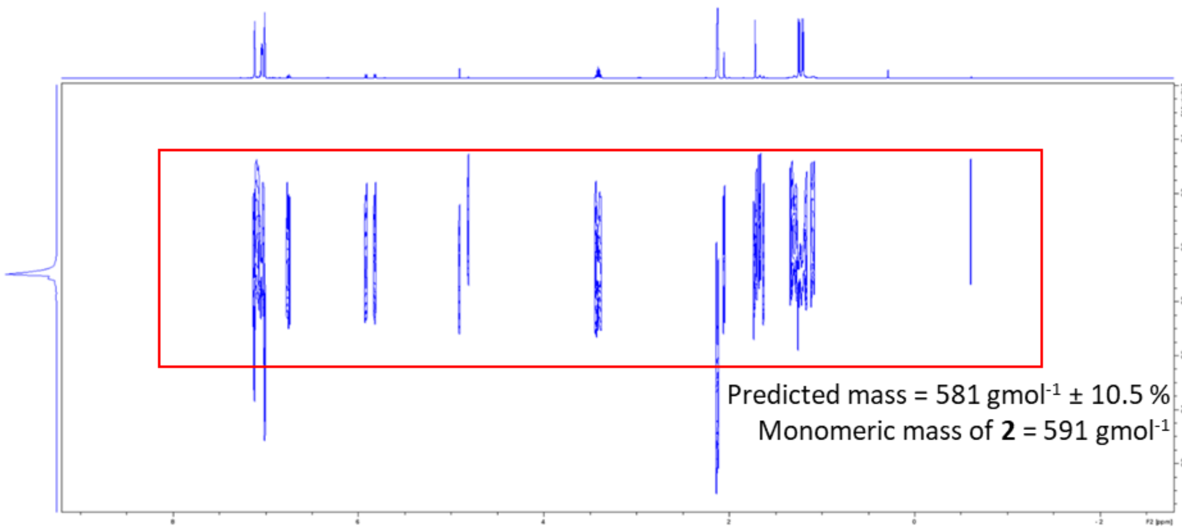

Figure S8: *DOSY NMR spectrum of **2** in $CD_3C_6D_5$ (298K)*

4. ROP of ε-CL in toluene/THF at RT to 60 °C catalysed by complexes **1 - 2**.

Table S1. *ε-CL ROP data*

Entry	Catalyst	Temperature	Time (h)	Conv.[a] (%)	M_n (kDa) *Calculated* [b]	M_n (kDa) *Observed* [c]	*Đ* [c]	*% Initiation efficiency* [d]
1	**1**	RT	24	6	-	-	-	-
2	**1**	60 °C	0.25	14	-	-	-	-
3	**1**	60 °C	0.50	33	-	-	-	-
4	**1**	60 °C	1	64	7.40	58.8	1.10	12.4
5	**1**	60 °C	2	93	-	-	-	-
6	**1**	60 °C	3	96	-	-	-	-
7	**1**	60 °C	4	97	11.10	121.11	2.24	9.1
8	**1**	60 °C	5	99	-	-	-	-
9	**2**	60 °C	0.33	79	9.04	68.10	1.33	6.6
10	**2**	60 °C	1	100	11.41	77.56	1.48	14.7
11	**2**	RT	1	2	0.22	9.46	-	2.5
12	**2**	RT	2	10	1.13	25.42	1.41	4.7
13	**2**	RT	3	23	2.57	40.46	1.41	6.7
14	**2**	RT	4	43	4.89	45.78	1.47	11.2
15	**2**	RT	5	53	6.13	43.29	1.85	14.9
16	**2**	RT	6	67	7.62	59.35	1.68	13.5
17[e]	**2**	RT	1	0	-	-	-	-
18[e]	**2**	RT	2	6	0.7	14.6	-	4.7
19[e]	**2**	RT	3	11	1.2	18.9	1.17	6.8
20[e]	**2**	RT	4	17	2.0	22.8	1.23	9.1
21[e]	**2**	RT	23	81	9.3	51.0	1.32	19.1

100:1 [ε-CL]:[cat], [ε-CL] = 0.87 M in toluene unless otherwise specified. ε-CL and the catalyst were both pre stirred in solvent for 5 minutes before mixing. a Conversion was calculated ex situ by ^{1}H NMR spectroscopy. b $M_{n\ calc}$ of polymers calculated from monomer conversion $M_{n\ calculated}$ = ([ε-CL]$_0$/[Cat]$_0$) x (% conversion of ε-CL) x 114.14, assuming 1 polymer chain per catalyst centre. c $M_{n\ observed}$ and Đ determined by size exclusion chromatography (SEC) using polystyrene standards in THF: values were corrected using a Mark-Houwink correction factor (0.56).[4] d calculated according to equations S1-3. e performed in THF ([ε-CL] = 0.87 M)

5. MALDI-ToF spectra

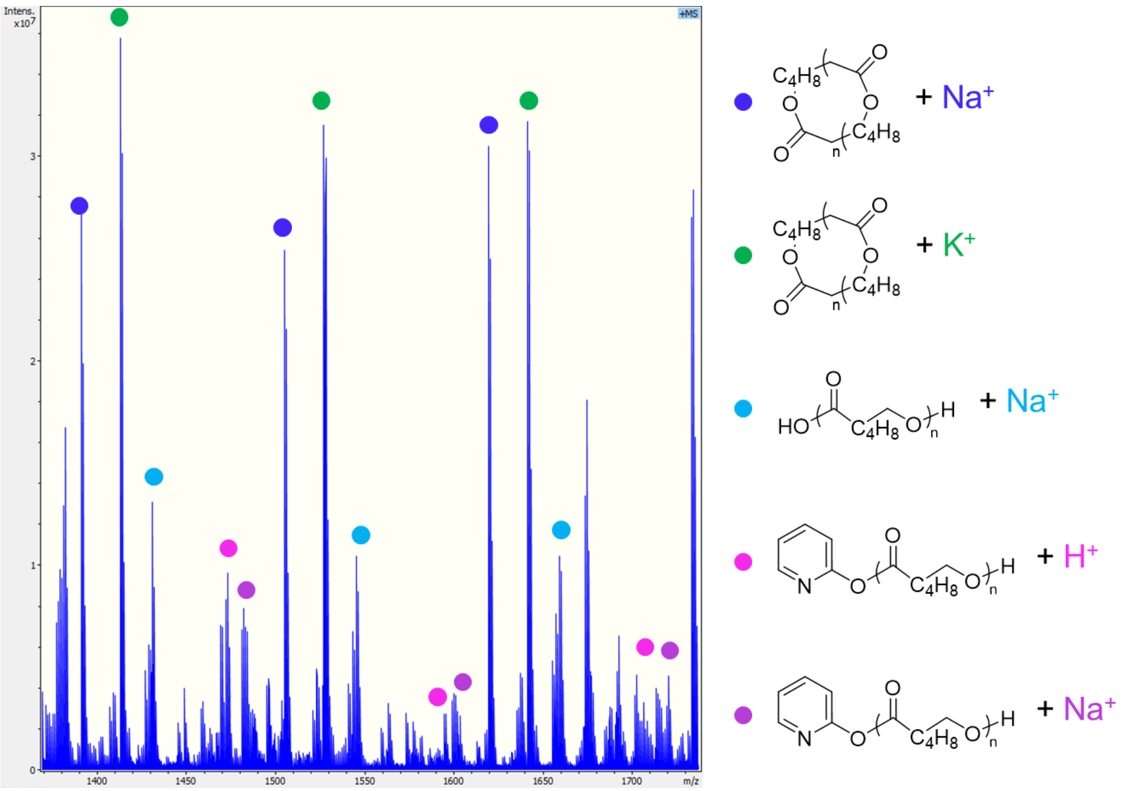

Figure S9. *MALDI-ToF MS analysis of crude PCL made in the presence of **1** after 4 h in toluene at 60 °C (table 2, entry 3).*

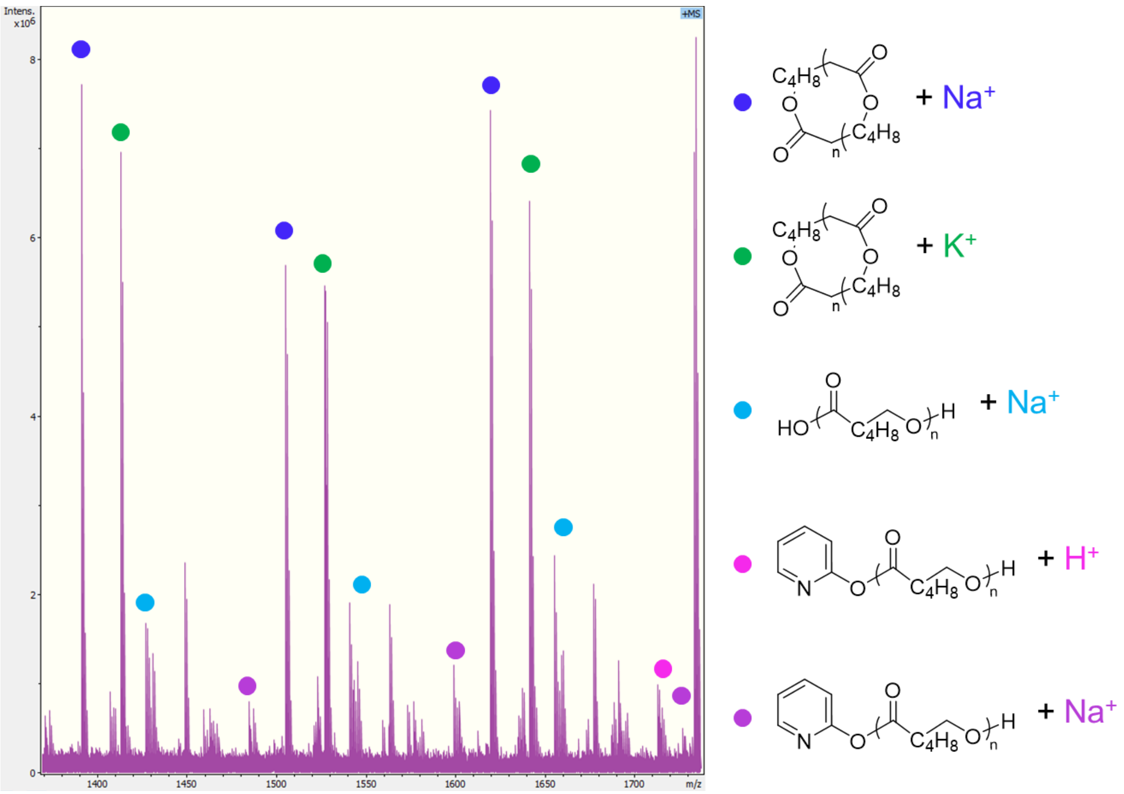

Figure S10. *MALDI-ToF MS analysis of crude PCL made in the presence of **1** after 24 h in toluene at 60 °C.*

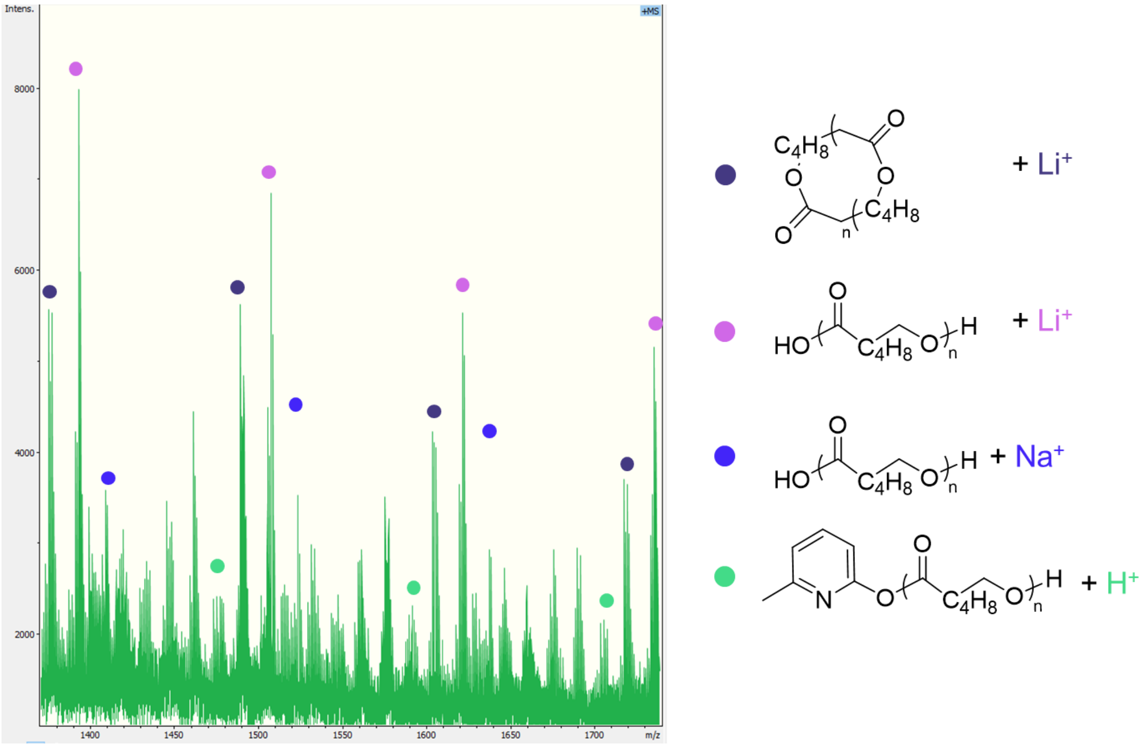

Figure S11. *MALDI-ToF MS analysis of crude PCL made in the presence of **2** after 2 h in toluene at RT (table 2, entry 7). MALDI-ToF preparation method b) was used.*

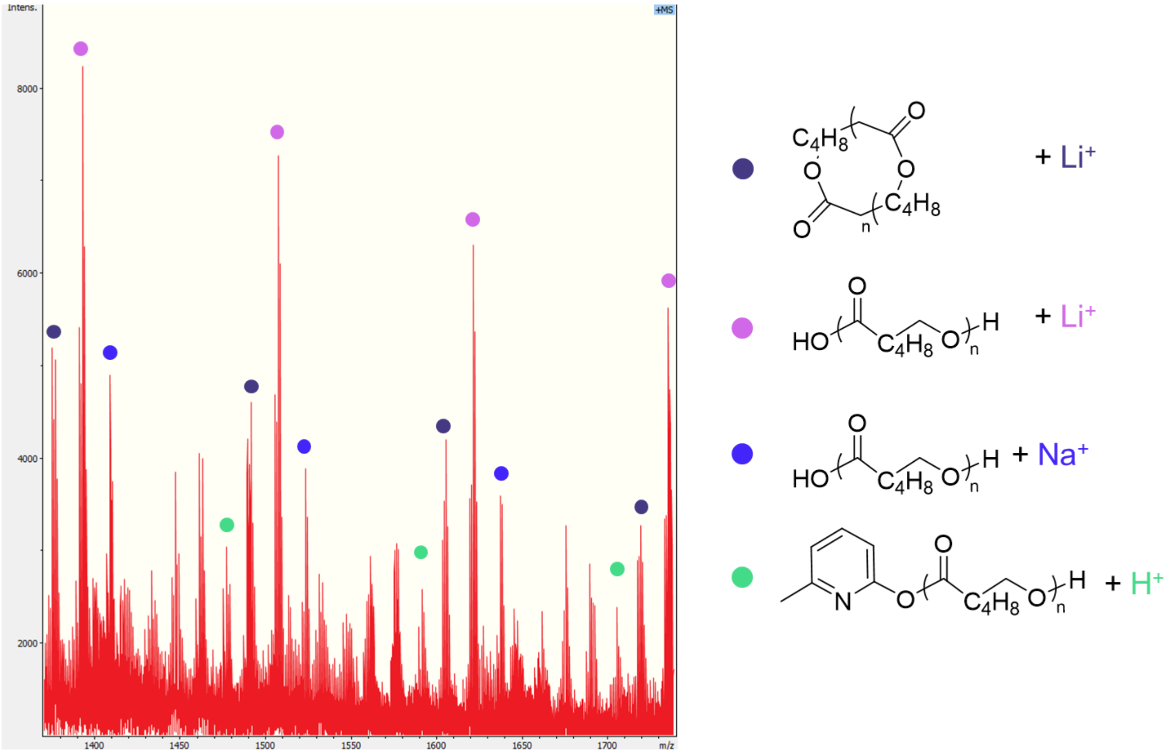

Figure S12. *MALDI-ToF MS analysis of crude PCL made in the presence of **2** after 2 h in THF at RT. MALDI-ToF preparation method b) was used.*

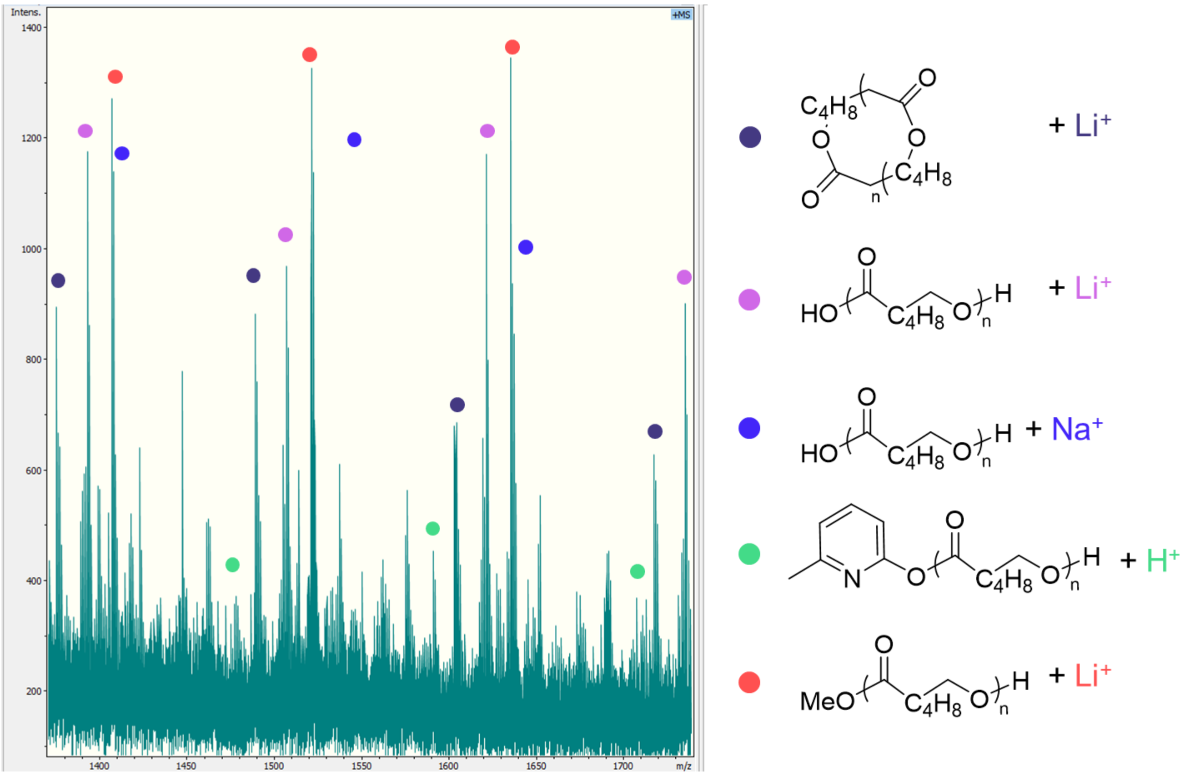

Figure S13. *MALDI-ToF MS analysis of purified PCL (cold MeOH/H$^+$) made in the presence of **2** after 4 h in toluene at RT. MALDI-ToF preparation method b) was used*

6. Proposed, simplified scheme for ring closure

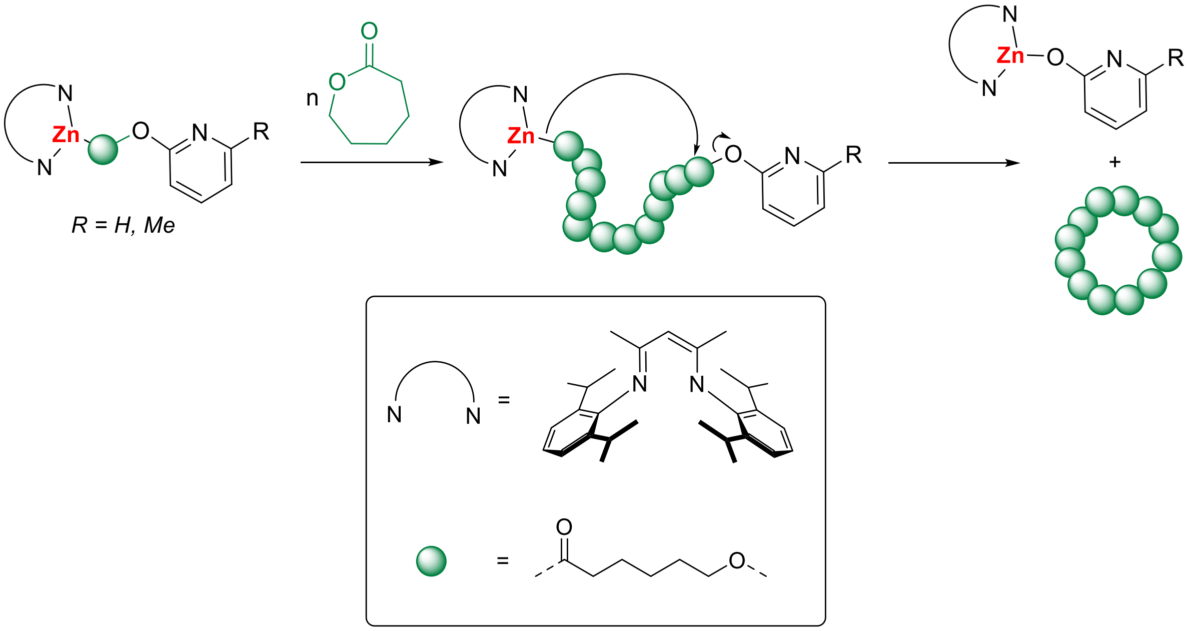

Figure S14. *Proposed ring-closure step using a simplified schematic: cyclisation of the PCL chain liberating the pyridonate anion to reform the catalytic species*

7. Example SEC traces

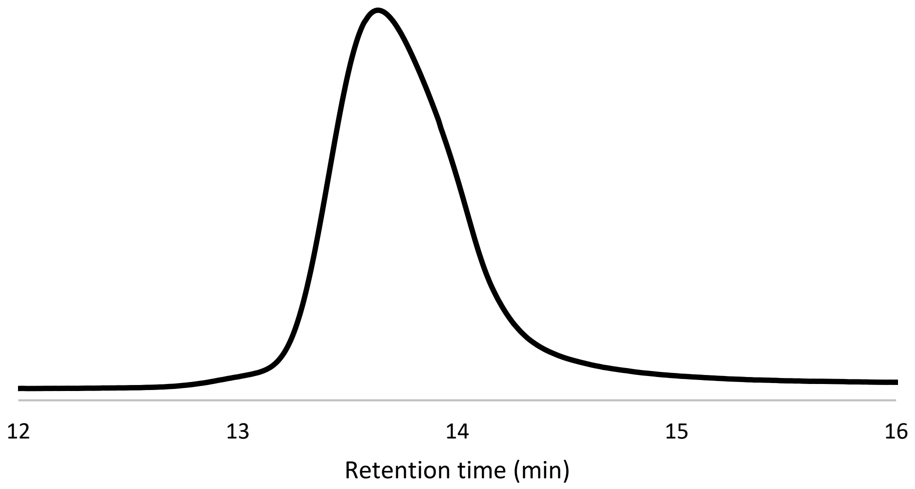

Figure S15. *Example SEC trace of crude PCL generated in the presence of **1** in toluene at 60 °C (1 h) (table 2, entry 2)*

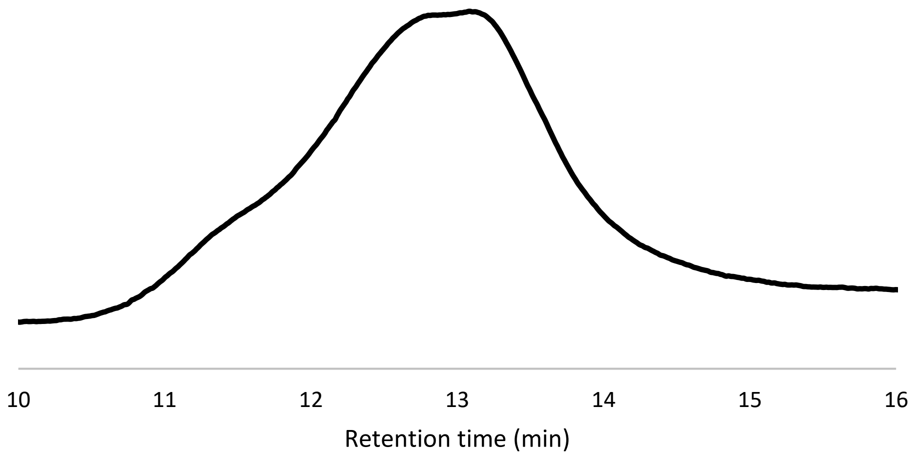

Figure S16. *Example SEC trace of crude PCL generated in the presence of **1** in toluene at 60 °C (4 h) (table 2, entry 3)*

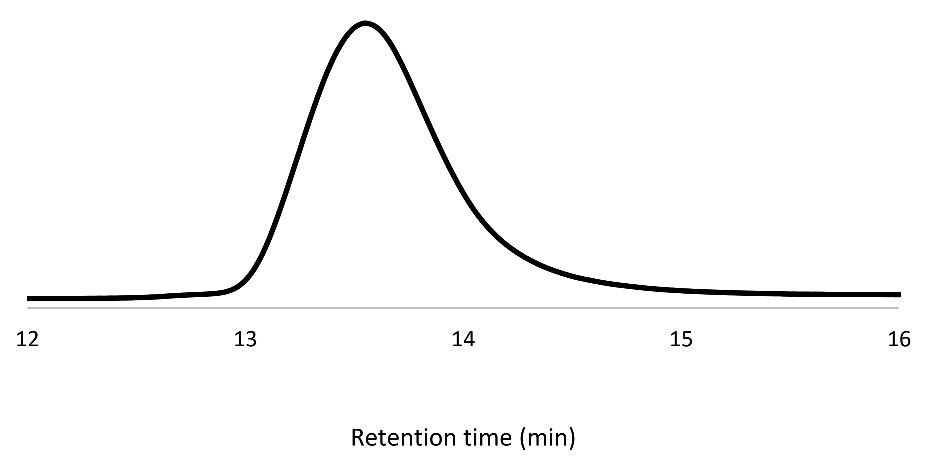

Figure S17. *Example SEC trace of crude PCL generated in the presence of* ***2*** *in toluene at RT (2 h) (table 2, entry 7)*

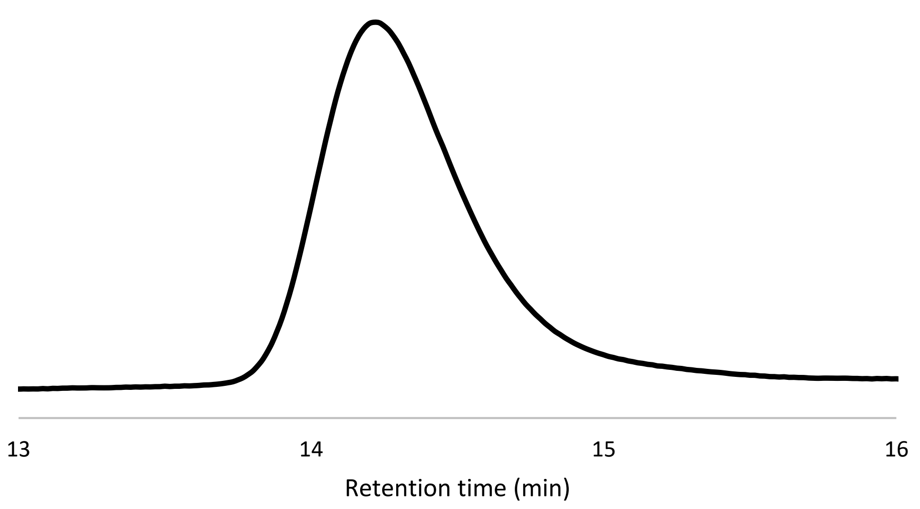

Figure S18. *Example SEC trace of crude PCL generated in the presence of* ***2*** *in THF at RT (2 h).*

8. Equations

Equation S1: Loading = [N x $M_{n, observed}$]/[Conversion x $MW_{\varepsilon CL}$]; $M_{n, observed}$ is the M_n value of the PCL obtained from SEC analysis ($gmol^{-1}$), Conversion= $[\varepsilon CL]_t/[\varepsilon CL]_0$; $MW_{\varepsilon CL}$= 114.14 $gmol^{-1}$; N = no. of ROP initiating groups *(1 in each instance)*.

Equation S2: $n_{cat} = n_{CL}$/Loading; n_{CL} = no. of mmol of εCL used; n_{cat} = no. of mmol of active catalyst, loading = value calculated using equation S1.

Equation S3: % active catalyst = $n_{cat}/n_{cat[0]}$; $n_{cat[0]}$ = no. of mmol of catalyst added; n_{cat} = value calculated using equation S2.[5]

9. Crystal refinement data

Table S2.** Crystal data and structure refinement for complex **1** (co-crystallised out of CH_2Cl_2) and **2

Complex	**1**	**2**
CCDC number	2285019	2285020
Empirical formula	$C_{34}H_{45}N_3OZn(CH_2Cl_2)_{0.5}$	$C_{35}H_{47}N_3OZn$
Formula weight	619.56	591.12
Temperature/K	120.01(11)	120.0(5)
Crystal system	monoclinic	triclinic
Space group	P21/c	P-1
a/Å	13.2654(6)	12.3962(3)
b/Å	16.8069(3)	16.5432(4)
c/Å	21.0819(11)	17.1649(4)
α/°	90	78.352(2)
β/°	135.126(9)	83.508(2)
γ/°	90	70.096(2)
Volume/Å^3	3316.2(4)	3237.86(14)
Z	4	4
ρ_{calc} g/cm^3	1.241	1.213
μ/mm^{-1}	0.851	0.789
F(000)	1316.0	1264.0
Crystal size/mm^3	0.439 × 0.158 × 0.09	0.425 × 0.216 × 0.073
Radiation	Mo Kα (λ = 0.71073)	Mo Kα (λ = 0.71073)
2Θ range for data collection/°	6.64 to 59.07	6.544 to 50.75
Index ranges	-17 ≤ h ≤ 18, -22 ≤ k ≤ 23, -29 ≤ l ≤ 26	-14 ≤ h ≤ 14, -19 ≤ k ≤ 19, -20 ≤ l ≤ 20
Reflections collected	72427	19098
Independent reflections	8388 [R_{int} = 0.0419, R_{sigma} = 0.0300]	19098 [R_{int} = ?, R_{sigma} = 0.1119]*
Data/restraints/parameters	8388/8/411	19098/30/744
Goodness-of-fit on F^2	1.032	0.916
Final R indexes [I>=2σ (I)]	R_1 = 0.0326, wR_2 = 0.0735	R_1 = 0.0503, wR_2 = 0.0932
Final R indexes [all data]	R_1 = 0.0469, wR_2 = 0.0795	R_1 = 0.0960, wR_2 = 0.1007
Largest diff. peak/hole / e Å^{-3}	0.59/-0.48	0.63/-0.51

non-merohedral twinning in the diffraction pattern of complex **2 was handled by CrysAlisPro, details are given in the accompanying CIF file.*

10. DOSY parameters and instrumentation

The Diffusion-Ordered Spectroscopy (DOSY) NMR experiments were performed at 298 K on a Bruker Ascend 2 channel instrument operating at a frequency of 500 MHz for proton resonance under TopSpin (version 3.2, Bruker Biospin, Karlsruhe) and equipped with a z-gradient DCH/5mm tuneable "CryoProbe"™ probe and a GRASP II gradient spectroscopy accessory providing a maximum gradient output of 53.5 G/cm (5.35G/cmA). Diffusion ordered NMR data was acquired using the Bruker pulse program dstebpgp3s with a spectral width of 10330 Hz (centred on 6.175 ppm) and 32768 data points. A relaxation delay of 2 s was employed along with a diffusion time (large delta) of 100 ms and a longitudinal eddy current delay (LED) of 5 ms. Bipolar gradients pulses (little delta/2) of 1.5 ms and homospoil gradient pulses of 0.6 ms were used. The gradient strengths of the 3 homospoil pulses were -13.17%, -17.13%, -15.37%. 16 experiments were collected with the bipolar gradient strength, initially at 5% (1st experiment), linearly increased to 95% (16th experiment). All gradient pulses were smooth-square shaped (SMSQ10.100) and after each application a recovery delay of 200 µs used. The experiment was run with 16 scans per increment, employing one stimulated echo with two spoiling gradients.

DOSY plots were generated by using the DOSY processing module of TopSpin. DOSY NMR spectra were formatted in TopSpin. Parameters were optimised empirically to find the best quality of data for presentation purposes. Diffusion coefficients were calculated by fitting intensity data to the Stejskal-Tanner expression. A calibration plot was formed through DOSY analysis of a range of standards spanning the molecular weight range of 136.24 to 622.67 g mol^{-1} [adamantane, 136.25; 2-phenylpyridine, 155.20; pyrene, 202.26; tri(*o*-tolyl)-phosphine, 304.37; tetraphenylphthaline, 432.50 and 2,2′-bis(diphenylphosphino)-1,1′-binaphthyl (BINAP), 622.67), Table S3]. Adamantane was run a total of four times and the average observed log(D) was used. Furthermore, adamantane was used as an internal standard for all other standards used. From the diffusion coefficients of the external standards, linear calibration graphs were obtained by plotting log(D) *vs* log(MW) (Figure S20). Following DOSY analysis of the product, the diffusion coefficient obtained for the signals corresponding to the product allowed an estimate of the MW of the species present in solution through use of the equation $y = mx + c$.

Table S3. *Diffusion coefficients of standards in toluene-d_8 solution compared to their molecular weights.*

			Gradient (m):		Intercept (c):				
**Calibration factors (from the plot equation y = mx + c):**			-0.6337		-7.3836			Uncorrected Adamantane	
**Calibrant**	**MW $_{real}$ (Da)**	**log *MW*$_{real}$**	**Observed log D**	**Normalised log D**	**MW$_{calc}$ (Da)**	**Difference**	**Error (%)**	log D_{obs}	Difference
Adamantane (Average)	136.25	2.13	-8.7678	-8.767875	152.87	16.63	12.20	-	-
2-Phenylpyridine	155.20	2.19	-8.7757	-8.7805	157.32	2.12	1.37	-8.763	-0.0048
Pyrene	202.26	2.31	-8.8288	-8.8235	190.80	-11.46	5.67	-8.7731	0.0053
Tri(*o*-tolyl)-phosphine	304.37	2.48	-8.8811	-8.8819	230.73	-73.64	24.19	-8.767	-0.0008
Tetraphenylnaphthaline	432.50	2.64	-9.0988	-9.103	508.91	76.41	17.67	-8.7636	-0.0042
BINAP	622.67	2.79	-9.1595	-9.1618	634.50	11.83	1.90	-8.7719	-0.0023
						**Average Error (%)**	10.5		

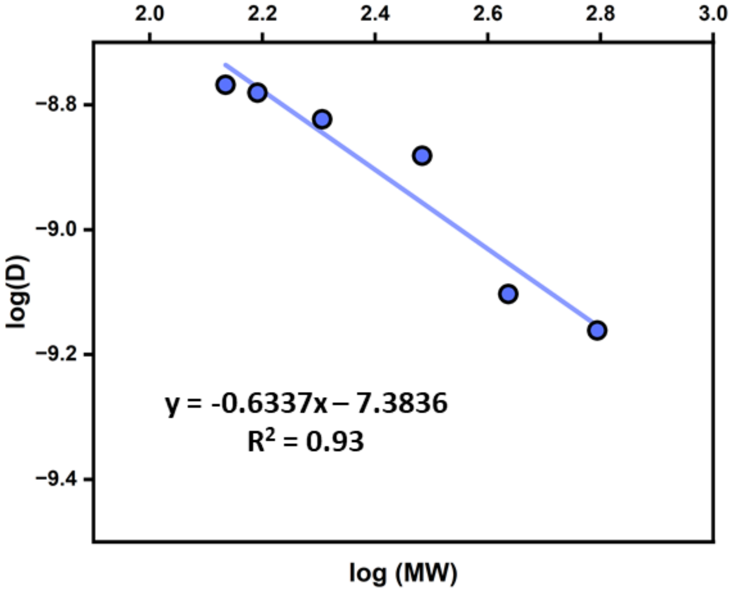

Figure S19. *Plot of Log(D) vs. log(MW) constructed from the ^{1}H DOSY NMR data of adamantane, 2-phenylpyridine, pyrene, tri(o-tolyl)-phosphine, tetraphenylphthaline and 2,2'-bis(diphenylphosphino)-1,1'-binaphthyl (BINAP) standards in toluene-d_8.*

11. Computational details for the structures of **1** and **2**

All of the calculations were performed using the Gaussian09 program[6] at B3PW91 level of theory[7,8] using LANL2DZ basis set for Zn atom and 6-311G(d,p) basis set for all other atoms. All geometry optimizations were full, with no restrictions. Stationary points located in the potential energy surface were characterized as minima (no imaginary frequencies) by vibrational analysis. Solvent effects of dichloromethane were introduced using the self-consistent field approach, by means of the integral equation formalism polarizable continuum model (IEFPCM).[9]

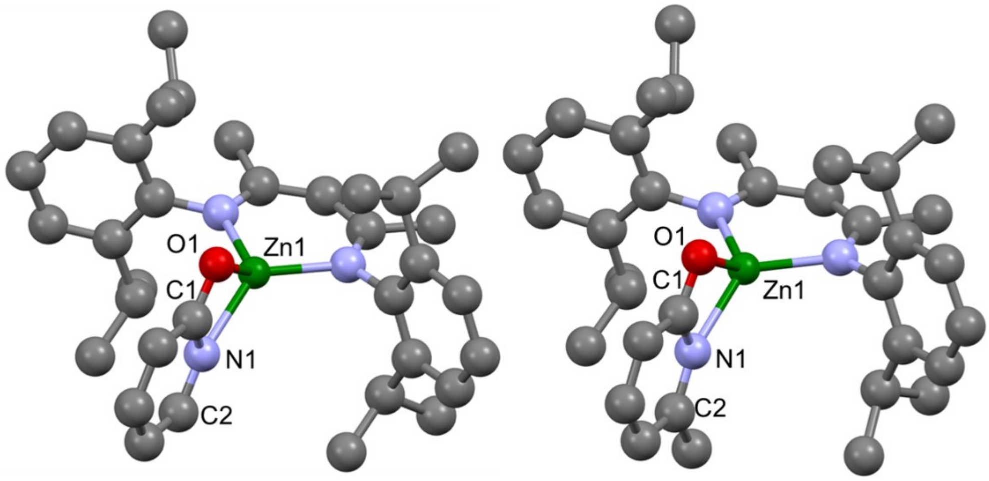

Figure S20. *Monomeric computed structures of **1** (left) and **2** (right)*

Table S4. *Bond lengths and angles calculated for monomeric **1** and **2***

Bond	***Bond length** / Å*	
	**1**	**2**
Zn1–N1	2.196	2.204
Zn1–O1	2.113	2.102
N1–C1	1.362	1.364
O1–C1	1.286	1.287
N1–C2	1.341	1.345
Bonds	***Bond angle** / °*	
N1-Zn1-O1	62.4	62.5
N1-C1-O1	115.1	114.9
Zn1-N1-C1	88.4	88.0
C1-O1-Zn1	94.1	94.6

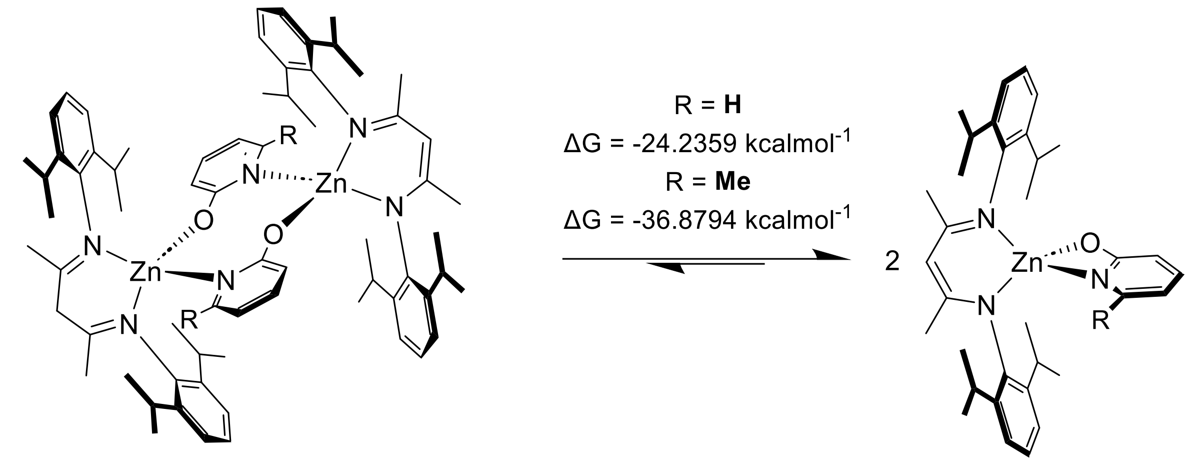

Figure S21: *Calculated gain in free energy upon dissolution of **1** and **2** into their monomeric species*

Coordinates calculated for monomers and dimers of **1** and **2**.

Monomeric **1**:

C	7.61030	17.42267	13.68231
C	8.20341	16.98149	14.88230
H	7.88521	17.52789	15.76071
C	9.08615	15.91971	15.14796
C	6.59083	18.52761	13.83928
H	6.58751	19.20799	12.98749
H	6.77398	19.09458	14.75223
H	5.58689	18.09687	13.91128
C	9.41524	15.68596	16.60317
H	8.97836	14.74267	16.94341
H	9.02652	16.48924	17.22783
H	10.49299	15.60510	16.75794
C	7.16294	17.42397	11.33766
C	5.88743	16.91772	11.00546
C	5.24943	17.40785	9.86433
H	4.26997	17.02279	9.59823
C	5.84096	18.37445	9.06395
H	5.32492	18.74806	8.18528
C	7.10208	18.85295	9.39035
H	7.56544	19.60369	8.75845
C	7.78808	18.38646	10.51342
C	5.20747	15.83258	11.82547
H	5.79220	15.69121	12.73781
C	3.78180	16.21487	12.24175
H	3.11665	16.29376	11.37633
H	3.75509	17.17250	12.76908
H	3.36510	15.45146	12.90591
C	5.21118	14.49778	11.06658
H	4.75542	13.71074	11.67654
H	6.22895	14.19045	10.81343
H	4.63501	14.57608	10.13849
C	9.16902	18.93971	10.83029
H	9.62410	18.27170	11.56639
C	10.08495	18.97007	9.60177
H	9.75039	19.70498	8.86338
H	10.12810	17.99476	9.11125
H	11.10112	19.24844	9.89777
C	9.08605	20.33540	11.46445
H	10.08734	20.70818	11.70308
H	8.50287	20.32807	12.38816
H	8.61790	21.04704	10.77635
C	10.46244	14.03820	14.62708
C	9.89674	12.77565	14.91195
C	10.74788	11.71801	15.23784
H	10.32339	10.74219	15.45318
C	12.12409	11.88745	15.29217
H	12.76845	11.05298	15.54989

C	12.66956	13.13199	15.01191
H	13.74675	13.26231	15.05367
C	11.86199	14.22032	14.67519
C	8.39758	12.52738	14.85077
H	7.90921	13.49279	14.69601
C	7.85132	11.93558	16.15639
H	6.76078	11.85749	16.10842
H	8.10681	12.55406	17.02134
H	8.24452	10.93072	16.33840
C	8.03783	11.63062	13.65806
H	8.51272	10.64830	13.75116
H	8.35744	12.07861	12.71378
H	6.95508	11.47446	13.61118
C	12.51538	15.55665	14.36103
H	11.71618	16.26737	14.13516
C	13.30590	16.11059	15.55372
H	12.67757	16.22311	16.44120
H	13.72149	17.09354	15.31065
H	14.14083	15.45400	15.81797
C	13.41598	15.45661	13.12338
H	14.26462	14.78931	13.30386
H	13.81769	16.44036	12.86009
H	12.86474	15.06991	12.26325
C	9.65537	14.04843	10.10036
C	9.93301	13.33616	8.90676
H	9.28300	12.51859	8.61937
C	11.02071	13.70635	8.14772
H	11.24435	13.16896	7.23100
C	11.84041	14.77270	8.54974
H	12.69890	15.08049	7.96654
C	11.51723	15.42579	9.72204
N	7.85886	16.91778	12.47898
N	9.61716	15.11927	14.22729
N	10.46453	15.07779	10.47576
O	8.67406	13.80895	10.89559
Zn	9.18343	15.36058	12.23691
H	12.11268	16.25831	10.08324

Monomeric **2**:

C	7.72967	17.49404	13.70167
C	8.34653	17.03965	14.88290
H	8.04553	17.57507	15.77451
C	9.17635	15.92994	15.13111
C	6.70137	18.58475	13.89745
H	6.68954	19.29135	13.06766
H	6.88408	19.12408	14.82702
H	5.70078	18.14539	13.95827
C	9.40329	15.61391	16.59194
H	8.64921	14.89728	16.93319
H	9.30211	16.51338	17.19995
H	10.37920	15.16393	16.77255
C	7.15337	17.47076	11.39063
C	5.91457	16.84867	11.11734
C	5.16293	17.29904	10.03052
H	4.21116	16.82501	9.81113
C	5.60478	18.33902	9.22566
H	5.00188	18.67931	8.38980
C	6.82731	18.93618	9.49577
H	7.17362	19.74718	8.86256
C	7.62203	18.51573	10.56441
C	5.38143	15.69096	11.94670
H	6.06515	15.54182	12.78589
C	3.98955	15.97976	12.52420
H	3.23712	16.06012	11.73338
H	3.96989	16.91125	13.09631
H	3.68034	15.16816	13.19010
C	5.36353	14.39410	11.12541
H	5.01275	13.55845	11.73967
H	6.36083	14.15019	10.75219
H	4.68670	14.48449	10.26936
C	8.95664	19.20185	10.80527
H	9.46680	18.64960	11.59900
C	9.84294	19.16322	9.55489
H	9.41278	19.75369	8.74019
H	9.97513	18.14068	9.19307
H	10.82950	19.58202	9.77572
C	8.78153	20.64957	11.28487
H	9.75691	21.10968	11.47249
H	8.20274	20.70597	12.20994
H	8.26704	21.25337	10.53043
C	10.45310	13.99181	14.57476
C	9.78356	12.78576	14.87594
C	10.54443	11.66590	15.21817
H	10.04062	10.73213	15.44756
C	11.92936	11.72107	15.26933
H	12.50207	10.84148	15.54557
C	12.57828	12.90607	14.95180

H	13.66219	12.94024	14.98161
C	11.86459	14.05010	14.59001
C	8.27111	12.65459	14.79678
H	7.85622	13.65686	14.66380
C	7.66160	12.06635	16.07525
H	6.56914	12.08013	16.01301
H	7.95663	12.63204	16.96338
H	7.96712	11.02645	16.22669
C	7.86749	11.82207	13.57127
H	8.26253	10.80346	13.64651
H	8.24438	12.26784	12.64725
H	6.77731	11.75309	13.49775
C	12.61053	15.32786	14.24114
H	11.91657	15.95309	13.67046
C	13.01089	16.11469	15.49705
H	12.14177	16.39561	16.09550
H	13.53854	17.03341	15.22125
H	13.67836	15.52019	16.12951
C	13.84231	15.07143	13.36672
H	14.62831	14.54526	13.91660
H	14.26775	16.02099	13.02989
H	13.59282	14.47765	12.48357
C	9.87656	14.18210	10.08388
C	10.20140	13.44472	8.92255
H	9.55415	12.63369	8.61224
C	11.34510	13.78050	8.23083
H	11.61878	13.22259	7.34005
C	12.16128	14.83013	8.66783
H	13.06462	15.09734	8.13299
C	11.79242	15.53503	9.80583
C	12.60327	16.69423	10.30404
H	12.36968	17.59674	9.73163
H	13.67215	16.50192	10.19135
H	12.38813	16.89718	11.35344
N	7.95361	17.00836	12.48361
N	9.70641	15.14995	14.19459
N	10.67788	15.21065	10.48518
O	8.85567	13.96410	10.83687
Zn	9.32699	15.49045	12.20344

Dimeric **1**:

C	-0.58935	4.59161	10.66666
C	-1.97252	4.38273	10.83175
H	-2.22908	3.42161	11.25884
C	-3.06895	5.25782	10.68625
C	1.37018	5.86622	10.23883
C	1.88781	6.58828	11.34029
C	3.26826	6.76637	11.44054
H	3.67526	7.30985	12.28658
C	4.13378	6.26698	10.47669
H	5.20450	6.41780	10.57148
C	3.61608	5.58116	9.38908
H	4.29167	5.20046	8.62929
C	2.24302	5.36453	9.25052
C	0.97493	7.14451	12.42153
H	-0.00962	7.27687	11.96325
C	0.80979	6.15946	13.58813
H	1.78025	5.93532	14.04360
H	0.36111	5.21781	13.26687
H	0.16557	6.58822	14.36239
C	1.43860	8.50352	12.95603
H	2.34006	8.41238	13.57010
H	0.66239	8.93962	13.59190
H	1.65345	9.20525	12.14657
C	1.74033	4.61185	8.03173
H	0.67154	4.43753	8.17189
C	1.90865	5.45496	6.76238
H	1.41072	6.42182	6.85877
H	1.48071	4.93759	5.89856
H	2.96612	5.64421	6.55434
C	2.41857	3.24723	7.85734
H	3.48252	3.35393	7.62413
H	1.95579	2.69893	7.03092
H	2.33703	2.63331	8.75839
C	-4.17310	7.30535	10.16878
C	-5.19255	7.18721	9.19905
C	-6.29591	8.04087	9.27694
H	-7.08399	7.95460	8.53527
C	-6.40730	8.99264	10.27798
H	-7.27654	9.64103	10.32417
C	-5.39235	9.11442	11.21757
H	-5.47851	9.86502	11.99582
C	-4.26617	8.29148	11.17950
C	-5.13490	6.18022	8.06266
H	-4.24863	5.56008	8.21362
C	-6.36273	5.25966	8.03281
H	-7.27186	5.81715	7.78633
H	-6.52990	4.76467	8.99275
H	-6.23640	4.48539	7.26962
C	-4.98146	6.88874	6.71102

H	-5.86479	7.49427	6.48534
H	-4.86321	6.15611	5.90664
H	-4.11259	7.54898	6.69696
C	-3.18539	8.43455	12.23900
H	-2.26004	8.04714	11.80065
C	-2.93783	9.88708	12.65634
H	-2.77583	10.53411	11.79070
H	-2.05195	9.94598	13.29548
H	-3.77467	10.29432	13.23217
C	-3.50086	7.58013	13.47547
H	-3.57998	6.51999	13.22692
H	-4.44759	7.89387	13.92774
H	-2.71440	7.68935	14.22897
C	0.29355	3.45134	11.12573
H	0.63597	2.88043	10.25670
H	-0.24954	2.77257	11.78290
H	1.18571	3.80930	11.64080
C	-4.38216	4.71517	11.20680
H	-5.01048	5.49856	11.63100
H	-4.21237	3.94635	11.96064
H	-4.94374	4.25963	10.38566
C	-0.20772	9.99554	8.89401
C	0.09272	12.02514	7.75784
H	0.18019	12.50923	6.78834
C	0.19131	12.75931	8.91810
H	0.36002	13.82800	8.88019
C	0.05484	12.06996	10.13167
H	0.11200	12.60393	11.07553
C	-0.14672	10.71112	10.12246
N	-0.04477	5.68332	10.13935
N	-3.01570	6.46519	10.13439
O	-0.31190	8.72279	8.85258
Zn	-1.28719	7.00382	9.07183
C	-1.08232	12.21485	4.20968
C	0.30070	12.42435	4.04412
H	0.55666	13.38542	3.61654
C	1.39769	11.55000	4.18993
C	-3.04103	10.93910	4.63835
C	-3.55831	10.21609	3.53731
C	-4.93861	10.03658	3.43763
H	-5.34539	9.49225	2.59203
C	-5.80429	10.53556	4.40158
H	-6.87488	10.38359	4.30721
C	-5.28691	11.22243	5.48865
H	-5.96262	11.60290	6.24845
C	-3.91400	11.44045	5.62667
C	-2.64521	9.66038	2.45600
H	-1.66062	9.52827	2.91426
C	-2.48027	10.64572	1.28960
H	-3.45078	10.86994	0.83428
H	-2.03151	11.58728	1.61096

H	-1.83614	10.21708	0.51519
C	-3.10842	8.30137	1.92107
H	-4.00966	8.39248	1.30668
H	-2.33188	7.86554	1.28541
H	-3.32345	7.59940	2.73028
C	-3.41161	12.19416	6.84493
H	-2.34299	12.36925	6.70441
C	-3.57895	11.35154	8.11475
H	-3.08033	10.38501	8.01866
H	-3.15113	11.86960	8.97821
H	-4.63621	11.16159	8.32318
C	-4.09083	13.55835	7.01876
H	-5.15467	13.45100	7.25218
H	-3.62832	14.10739	7.84484
H	-4.00985	14.17187	6.11738
C	2.50330	9.50351	4.70852
C	3.52265	9.62297	5.67820
C	4.62651	8.76991	5.60096
H	5.41449	8.85713	6.34262
C	4.73853	7.81754	4.60055
H	5.60818	7.16966	4.55485
C	3.72373	7.69453	3.66097
H	3.81044	6.94349	2.88320
C	2.59701	8.51677	3.69846
C	3.46432	10.63072	6.81389
H	2.57770	11.25025	6.66246
C	4.69162	11.55199	6.84323
H	5.60103	10.99521	7.09025
H	4.85864	12.04635	5.88293
H	4.56474	12.32675	7.60582
C	3.31114	9.92300	8.16598
H	4.19492	9.31835	8.39225
H	3.19209	10.65608	8.96983
H	2.44279	9.26211	8.18023
C	1.51632	8.37232	2.63905
H	0.59069	8.75924	3.07721
C	1.26985	6.91940	2.22241
H	1.10843	6.27265	3.08837
H	0.38395	6.85951	1.58338
H	2.10695	6.51254	1.64670
C	1.83109	9.22641	1.40217
H	1.90962	10.28669	1.65028
H	2.77795	8.91305	0.94990
H	1.04458	9.11641	0.64883
C	-1.96594	13.35453	3.75053
H	-2.30796	13.92576	4.61950
H	-1.42350	14.03315	3.09265
H	-2.85831	12.99601	3.23623
C	2.71050	12.09321	3.66897
H	3.33916	11.31004	3.24486
H	2.54016	12.86172	2.91494

H	3.27200	12.54928	4.48988
C	-1.46212	6.81127	5.98335
C	-1.76472	4.78110	7.11800
H	-1.85310	4.29649	8.08714
C	-1.86307	4.04784	5.95712
H	-2.03251	2.97924	5.99412
C	-1.72543	4.73798	4.74414
H	-1.78238	4.20470	3.79987
C	-1.52297	6.09671	4.75436
H	-1.42013	6.66026	3.83648
N	-1.62620	11.12304	4.73748
N	1.34532	10.34285	4.74237
N	-1.56021	6.11401	7.15078
O	-1.35678	8.08386	6.02639
Zn	-0.38293	9.80306	5.80450
H	-0.24876	10.14808	11.04075
N	-0.11091	10.69203	7.72600

Dimeric form of **2**:

C	-0.51046	4.76573	10.85601
C	-1.88979	4.59667	11.08217
H	-2.14136	3.69231	11.62289
C	-2.99148	5.42877	10.80949
C	1.44767	5.93132	10.18682
C	2.02016	6.66261	11.25651
C	3.40840	6.79681	11.31344
H	3.85557	7.34021	12.13918
C	4.23126	6.25453	10.33619
H	5.30859	6.36974	10.40064
C	3.66024	5.57364	9.27285
H	4.30002	5.15671	8.50106
C	2.27785	5.39683	9.17839
C	1.17250	7.28846	12.35421
H	0.15452	7.36831	11.96168
C	1.11787	6.41255	13.61471
H	2.12346	6.25918	14.02074
H	0.68147	5.43252	13.41690
H	0.51404	6.89722	14.38864
C	1.65086	8.69532	12.73225
H	2.60226	8.66759	13.27227
H	0.92167	9.17407	13.39324
H	1.77941	9.32753	11.85133
C	1.72577	4.61081	8.00366
H	0.63944	4.60893	8.09828
C	2.07377	5.26874	6.66442
H	1.75122	6.31182	6.63341
H	1.58396	4.73807	5.84278
H	3.15187	5.25027	6.47904
C	2.19879	3.15103	8.01790
H	3.28371	3.08622	7.88594
H	1.73167	2.59062	7.20185
H	1.94877	2.65272	8.95780
C	-4.10147	7.41543	10.12853
C	-5.06318	7.34032	9.09802
C	-6.18631	8.16898	9.16047
H	-6.93117	8.11055	8.37276
C	-6.37610	9.05342	10.20993
H	-7.26141	9.68032	10.24690
C	-5.41644	9.13688	11.20952
H	-5.56207	9.83792	12.02447
C	-4.26960	8.34182	11.18716
C	-4.94009	6.37491	7.93293
H	-4.00268	5.83040	8.05918
C	-6.08584	5.35342	7.91323
H	-7.04763	5.84327	7.73053
H	-6.16598	4.81037	8.85806
H	-5.93107	4.62220	7.11342
C	-4.86985	7.11284	6.59110

H	-5.79680	7.65890	6.39067
H	-4.72309	6.39902	5.77452
H	-4.04458	7.82754	6.56407
C	-3.25151	8.47101	12.31114
H	-2.30366	8.08430	11.92434
C	-3.03171	9.92404	12.74718
H	-2.84610	10.57887	11.89328
H	-2.17035	9.98579	13.41930
H	-3.89342	10.31686	13.29573
C	-3.63196	7.62487	13.53520
H	-3.68001	6.56060	13.30132
H	-4.60811	7.93147	13.92593
H	-2.89410	7.75788	14.33293
C	0.35902	3.66998	11.43814
H	0.43414	2.85549	10.70901
H	-0.08429	3.25615	12.34467
H	1.37131	4.00650	11.65495
C	-4.31497	4.90116	11.32534
H	-5.02112	5.69432	11.56673
H	-4.17056	4.27107	12.20346
H	-4.77435	4.28197	10.54742
C	-0.28051	9.89445	8.97049
C	0.01856	12.11974	8.25343
C	-0.04724	12.59392	9.55149
H	0.07978	13.65185	9.74455
C	-0.27956	11.68860	10.59148
H	-0.35240	12.03985	11.61629
C	-0.40063	10.35235	10.30775
N	0.02777	5.75229	10.14439
N	-2.94491	6.57046	10.12469
O	-0.27209	8.65063	8.67578
Zn	-1.25292	6.90196	8.89821
C	-1.15917	12.04086	4.01953
C	0.22025	12.20989	3.79410
H	0.47210	13.11396	3.25302
C	1.32189	11.37800	4.06785
C	-3.11763	10.87503	4.68734
C	-3.68891	10.14382	3.61689
C	-5.07705	10.00910	3.55876
H	-5.52329	9.46573	2.73250
C	-5.90101	10.55085	4.53538
H	-6.97823	10.43520	4.46994
C	-5.33121	11.23176	5.59933
H	-5.97183	11.64829	6.37065
C	-3.94897	11.40904	5.69507
C	-2.84010	9.51851	2.51971
H	-1.82255	9.43833	2.91330
C	-2.78394	10.39505	1.25969
H	-3.78907	10.54880	0.85267
H	-2.34753	11.37487	1.45837
H	-2.17940	9.91057	0.48619

C	-3.31821	8.11194	2.14026
H	-4.26865	8.14021	1.59859
H	-2.58803	7.63330	1.48029
H	-3.44848	7.47940	3.02068
C	-3.39833	12.19514	6.87042
H	-2.31195	12.19771	6.77666
C	-3.74691	11.53677	8.20929
H	-3.42335	10.49402	8.24048
H	-3.25828	12.06782	9.03140
H	-4.82517	11.55422	8.39379
C	-3.87220	13.65464	6.85605
H	-4.95727	13.71878	6.98714
H	-3.40606	14.21517	7.67258
H	-3.62171	14.15327	5.91644
C	2.43180	9.39217	4.75133
C	3.39285	9.46849	5.78236
C	4.51616	8.63995	5.72137
H	5.26052	8.69921	6.50947
C	4.70681	7.75467	4.67277
H	5.59230	7.12798	4.63688
C	3.74776	7.67005	3.67269
H	3.89406	6.96830	2.85847
C	2.60064	8.46474	3.69375
C	3.26890	10.43513	6.94635
H	2.33142	10.97931	6.81904
C	4.41439	11.45692	6.96548
H	5.37621	10.96748	7.14909
H	4.49478	11.99899	6.02011
H	4.25908	12.18894	7.76445
C	3.19813	9.69866	8.28898
H	4.12510	9.15305	8.49051
H	3.05079	10.41339	9.10466
H	2.37299	8.98384	8.31636
C	1.58294	8.33393	2.56959
H	0.63503	8.72133	2.95550
C	1.36304	6.88019	2.13596
H	1.17667	6.22698	2.99093
H	0.50208	6.81743	1.46342
H	2.22500	6.48617	1.58868
C	1.96385	9.17814	1.34435
H	2.01207	10.24275	1.57669
H	2.94003	8.87079	0.95427
H	1.22614	9.04410	0.54666
C	-2.02853	13.13643	3.43691
H	-2.10591	13.95001	4.16682
H	-1.58394	13.55169	2.53166
H	-3.04010	12.79917	3.21784
C	2.64554	11.90514	3.55195
H	3.35169	11.11173	3.31139
H	2.50139	12.53452	2.67328
H	3.10473	12.52492	4.32950

C	-1.39153	6.91377	5.90790
C	-1.69389	4.68851	6.62352
C	-1.62967	4.21525	5.32501
H	-1.75835	3.15764	5.13123
C	-1.39657	5.12098	4.28558
H	-1.32486	4.77038	3.26046
C	-1.27310	6.45684	4.57022
H	-1.11230	7.19470	3.79676
N	-1.69781	11.05449	4.73117
N	1.27514	10.23694	4.75363
N	-1.54220	6.00542	6.92039
O	-1.39689	8.15724	6.20415
Zn	-0.41766	9.90625	5.97940
H	-0.56052	9.61479	11.08170
N	-0.13106	10.80235	7.95739
C	-1.98263	3.73448	7.74145
H	-1.33291	3.89986	8.60127
H	-3.01036	3.86000	8.09620
H	-1.86620	2.70257	7.40788
C	0.30700	13.07320	7.13497
H	1.33560	12.94948	6.78208
H	-0.34097	12.90545	6.27429
H	0.18780	14.10524	7.46717

12. References

1. M. P. Blake, N. Kaltsoyannis and P. Mountford, *Chem. Commun*., 2015, **51** (26), 5743–5746.

2. A. P. Dove, V. C. Gibson, E. L. Marshall, A. J. P. White and D. J. Williams, *Dalton Trans*., 2004, **4**, 570–578.

3. P. Ni, L. Yang, Y. Shen, L. Zhang, Y. Ma, M. Sun, R. Cheng, and J. Ye, *J. Org. Chem.,* 2022, **87** (19), 12677–12687.

4. A. Kowalski, A. Duda and S. Penczek, *Macromolecules,* 1998, **31** (7), 2114–2122.

5. A. Thevenon , C. Romain , M. S. Bennington , A. J. White , H. J. Davidson , S. Brooker and C. K. Williams, *Angew. Chem., Int. Ed.*, 2016, **55**, 8680 —8685.

6. Gaussian 09, Revision E.01, M. J. Frisch, G. W. Trucks, H. B. Schlegel, G. E. Scuseria, M. A. Robb, J. R. Cheeseman, G. Scalmani, V. Barone, B. Mennucci, G. A. Petersson, H. Nakatsuji, M. Caricato, X. Li, H. P. Hratchian, A. F. Izmaylov, J. Bloino, G. Zheng, J. L. Sonnenberg, M. Hada, M. Ehara, K. Toyota, R. Fukuda, J. Hasegawa, M. Ishida, T. Nakajima, Y. Honda, O. Kitao, H. Nakai, T. Vreven, J. A. Montgomery, Jr., J. E. Peralta, F. Ogliaro, M. Bearpark, J. J. Heyd, E. Brothers, K. N. Kudin, V. N. Staroverov, T. Keith, R. Kobayashi, J. Normand, K. Raghavachari, A. Rendell, J. C. Burant, S. S. Iyengar, J. Tomasi, M. Cossi, N. Rega, J. M. Millam, M. Klene, J. E. Knox, J. B. Cross, V. Bakken, C. Adamo, J. Jaramillo, R. Gomperts, R. E. Stratmann, O. Yazyev, A. J. Austin, R. Cammi, C. Pomelli, J. W. Ochterski, R. L. Martin, K. Morokuma, V. G. Zakrzewski, G. A. Voth, P. Salvador, J. J. Dannenberg, S. Dapprich, A. D. Daniels, O. Farkas, J. B. Foresman, J. V. Ortiz, J. Cioslowski, and D. J. Fox, Gaussian, Inc., Wallingford CT, 2013.

7. Becke, A. D., *J. Chem. Phys.,* 1993, **98** (7), 5648–5652.

8. Electronic Density Functional Theory: Recent Progress and New Directions; Dobson, J. F., Vignale, G., Das, M. P., Eds.; Springer US: Boston, MA, 1998.

9. B. Mennucci, E. Cancès and J. Tomasi, *J. Phys. Chem. B,* 1997, **101** (49), 10506–10517.
